# Killing hepatocellular carcinoma in the NAFLD/NASH stage: a comprehensive perspective on targeting regulated cell death

**DOI:** 10.1038/s41420-025-02558-x

**Published:** 2025-06-19

**Authors:** Jianxin Xi, Shuangyin Lei, Jie Chen, Jiahui Liu, Chenhao Shan, Xun Sun, Qianqian Zheng, Xiaoju Shi

**Affiliations:** 1 Department of Hepatobiliary and Pancreatic Surgery, General Surgery Center, The First Hospital of Jilin University, Changchun, China; 2 Department of Neurology, The First Hospital of Jilin University, Changchun, China; 3 Department of Radiology, The First Hospital of Jilin University, Changchun, China; 4 Basic Medicine College, China Medical University, Shenyang, China; 5 Department of Immunology, Basic Medicine College, China Medical University, Shenyang, China; 6 Department of Pathophysiology, Basic Medicine College, China Medical University, Shenyang, China

**Keywords:** Non-alcoholic steatohepatitis, Cell death

## Abstract

Nonalcoholic steatohepatitis (NASH) has been identified as a significant risk factor contributing to the rising incidence of hepatocellular carcinoma (HCC). With the evolving epidemiological characteristics of NASH, the incidence of NASH-related HCC has increased substantially. Recent advances in the study of regulated cell death (RCD) mechanisms have uncovered their roles in the pathogenesis of NAFLD/NASH and associated HCC, offering novel insights and directions for targeted therapeutic strategies. Although numerous studies have highlighted the critical role of RCD mechanisms in NAFLD/NASH and related HCC, significant challenges remain in developing effective targeted therapies and translating them into clinical applications. This review aims to summarize the current progress in understanding the role of RCD in NAFLD/NASH and associated HCC, explore potential therapeutic strategies and clinical applications, and provide new perspectives and therapeutic targets for treating NAFLD/NASH. Ultimately, the goal is to control disease progression at the NAFLD/NASH stage and prevent its progression to HCC.

## Facts

- As the epidemiological characteristics of NAFLD/NASH evolve, it is expected to become the leading risk factor for the development of HCC in the future.
- RCD plays a critical role in the pathophysiological mechanisms of NAFLD/NASH and facilitates the progression from NAFLD/NASH to HCC.
- Novel therapeutic strategies based on RCD mechanisms hold promise as effective approaches to control NAFLD/NASH and its progression to HCC. Moreover, targeted drug development against RCD pathways has made significant progress.

## Open questions

- The interplay between different types of cell death mechanisms in NAFLD/NASH and HCC is not yet fully understood. Do different RCD pathways play dominant roles at various stages of the disease? Is there a regulatory interplay among these mechanisms?
- Although RCD mechanisms have demonstrated potential in animal models, translating these foundational findings into effective clinical treatments remains a significant challenge. What is the clinical efficacy of current targeted therapeutic strategies in NASH-related HCC?
- The clinical presentation and pathological progression of NAFLD/NASH exhibit significant individual variability. How can personalized RCD-targeted therapeutic strategies be tailored based on a patient’s specific pathological conditions, such as the degree of liver fibrosis and patterns of fat accumulation?
- In addition to RCD mechanisms, the progression of NAFLD/NASH and HCC may be influenced by multiple factors, including genetic, immune, and environmental factors. Do these factors interact with RCD mechanisms to promote the transition from NASH to HCC? Is it necessary to consider these multifaceted factors in developing new therapeutic approaches?

## Introduction

Non-alcoholic steatohepatitis (NASH) was initially reported in 1980 as a specific phase within the spectrum of nonalcoholic fatty liver disease (NAFLD), secondary to simple hepatic steatosis, representing its inflammatory subtype. The hallmark pathological manifestations of NASH include steatosis, hepatocyte ballooning, lobular inflammation, and fibrosis [1]. Closely associated with complex metabolic disorders, NASH progresses through chronic inflammatory processes that promote and sustain a pro-oncogenic environment, leading to hepatocyte injury, thereby emerging as a principal etiological factor of terminal liver diseases, including hepatocellular carcinoma (HCC) and liver failure [2, 3]. NASH-HCC follows a unique pathological trajectory. It begins with simple hepatic fat accumulation. Over time, this can develop into NASH. As the disease advances, it may lead to cirrhosis. In some cases, this ultimately progresses to HCC. Although most HCC cases in NASH arise secondary to cirrhosis, approximately 30%–40% of tumors occur in patients without cirrhosis, making NASH the leading cause of non-cirrhotic HCC [4–6]. Due to the complexity and diversity of the drivers involved in disease development and progression, NASH-HCC has become a major public health issue. Typically, patients are diagnosed at an advanced stage, and systemic therapies are recommended as the standard treatment [7]. However, compared to virus-related HCC subtypes, NASH-HCC demonstrates a poor response to immune checkpoint inhibitors, the gold standard for the treatment of advanced HCC, primarily due to differences in the tumor microenvironment [8]. Consequently, a thorough comprehension of NASH pathogenesis and the identification of key therapeutic targets are imperative. Hepatocyte death is a key event and pathogenic mechanism in the transition of NASH to HCC. Recent advancements in understanding the diverse forms of regulated cell death (RCD) and their mechanistic roles in liver disease models have provided significant insights [9].

**Fig. 1:**
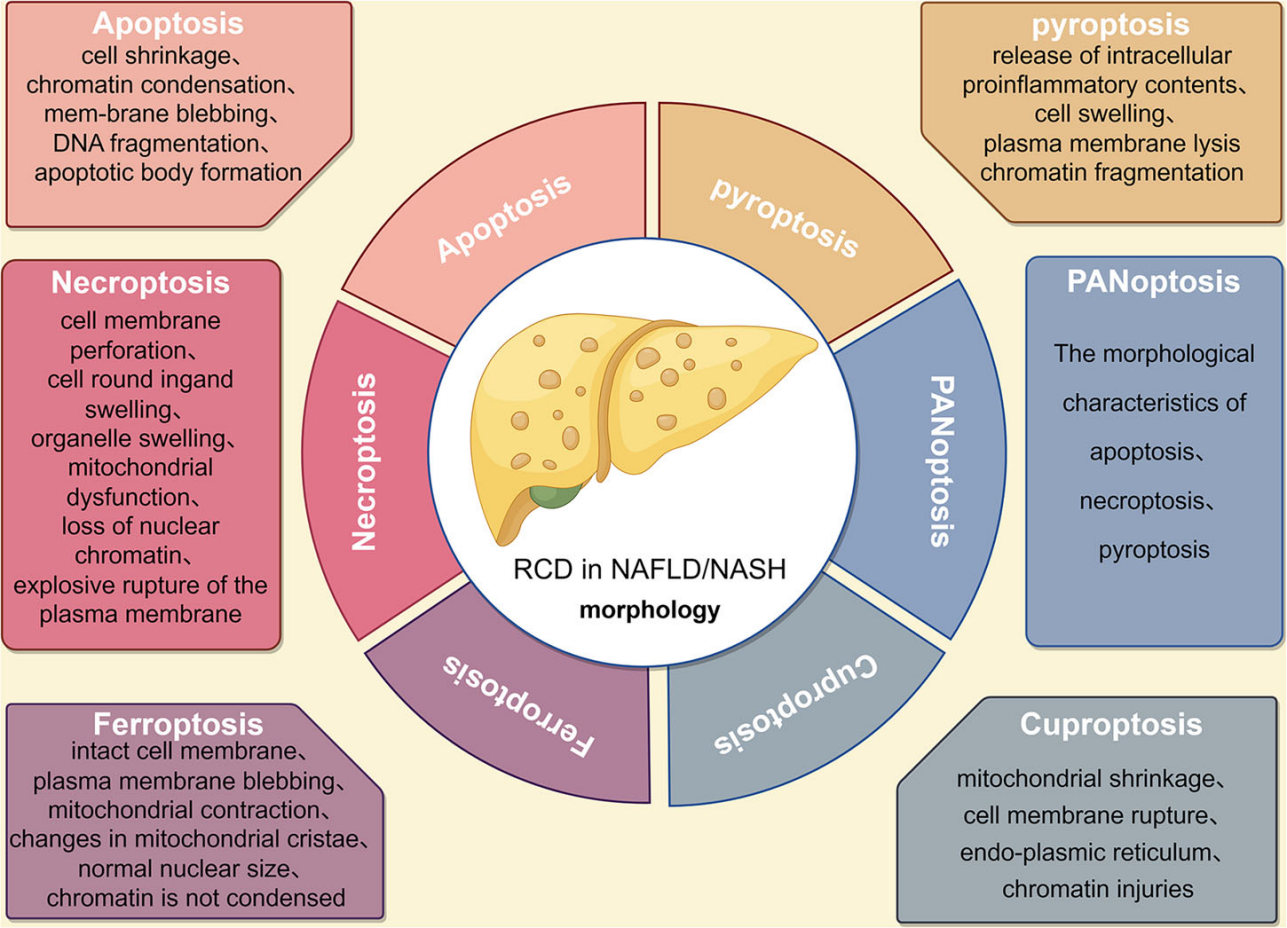
Morphological characteristics of different RCD types.

RCD, alternatively termed as programmed cell death (PCD), is a genetically encoded mechanism through which cells undergo autonomous and orderly death to maintain internal homeostasis, unlike accidental cell death [10, 11]. The currently known types of RCD include apoptosis, pyroptosis, necroptosis, ferroptosis, cuproptosis, parthanatos, autophagy-dependent cell death, entosis, NETosis, lysosome-dependent cell death, alkaliptosis, and oxeiptosis [11]. These distinct cell death pathways can be systematically categorized according to their unique morphological characteristics (Fig. 1) [12–16]. Each type of RCD exhibits immunoregulatory features, spanning a spectrum from anti-inflammatory and tolerogenic responses to pro-inflammatory and immunogenic reactions [10, 17]. In liver diseases, inflammation resulting from hepatocyte death can lead to fibrosis, thereby promoting disease progression. RCD plays a crucial role in determining the severity and outcomes of liver injuries. Various types of cell death may coexist with overlapping features and crosstalk [9]. While the evasion of RCD is a hallmark of cancer, blocking RCD pathways may exhibit anticancer therapeutic effects, thereby influencing cancer progression and treatment response [18].

Currently, there are no approved pharmacological interventions specifically targeting NAFLD/NASH or NAFLD/NASH-HCC. The critical role of RCD in NAFLD/NASH and the crosstalk between different RCD pathways provide new perspectives and opportunities for controlling NAFLD/NASH and preventing its progression to HCC. The key features and mechanisms of various forms of RCD, as well as recent findings and applications in NAFLD/NASH, are briefly reviewed in this article. The goal was to identify potential therapeutic targets and develop innovative treatment strategies for NAFLD/NASH, thereby transforming our approach to HCC prevention in the context of NAFLD/NASH.

## Pathogenic mechanisms of NAFLD/NASH-HCC

### Pathogenic mechanisms of NAFLD/NASH and its promotion of HCC development

NAFLD is a leading cause of chronic liver disease worldwide. It is associated with low-grade chronic inflammation and is closely related to metabolic syndromes, including obesity, insulin resistance (IR), type 2 diabetes (T2DM), hypertension, and dyslipidemia. In addition to being a complex metabolic disorder, NAFLD includes numerous diseases, ranging from the non-progressive form of NAFL to NASH, which eventually progresses to cirrhosis and liver cancer [19]. The dynamic nature of this progression and multiple triggering factors make it difficult to describe the exact mechanisms underlying NAFLD. To understand the pathogenesis of NAFLD, Day et al. proposed the classic “two-hit” hypothesis in 1998. According to this theory, the first “hit” occurs due to the accumulation of lipids like triglycerides, followed by the second “hit,” where free radicals produced by cytokines and inflammatory mediators exacerbate the damage, exacerbating lipid metabolism abnormalities [20]. As research has progressed, it has become evident that the pathological process of NAFLD is likely to be more complex, involving multiple factors acting in parallel or sequentially to promote the disease. This led to the development of the “multiple hits” hypothesis. This theory includes various factors, including lipid accumulation, IR, adipose tissue dysfunction, diet and nutrition, immune responses, changes in the gut microbiome, and genetic and epigenetic factors [21].

Lipid metabolism and IR are key factors in NAFLD development. Excessive intake of carbohydrates and fats leads to energy accumulation in the liver and white adipose tissue (WAT) in the form of triglycerides. Moreover, the excess glucose-induced enhancement of de novo lipogenesis (DNL) in the liver causes an influx of free fatty acids (FFAs) into the liver. This process can activate macrophages through adipokines, lipids, or lipid metabolites, leading to a pro-inflammatory state that promotes IR [22]. IR inhibits the action of insulin, increasing hepatic DNL and promoting lipolysis in peripheral WAT, thereby increasing FFA flux into the liver. This imbalance in lipid metabolism creates a lipotoxic microenvironment, where reactive oxygen species (ROS) are excessively produced due to the oxidation of FFAs, a key factor in driving cellular stress. The resulting oxidative and endoplasmic reticulum (ER) stress disrupts homeostasis, activating a cascade of inflammatory and immune responses that lead to hepatocyte death. These processes are interdependent, creating a vicious cycle [22]. Additionally, gut microbiota, epigenetic factors (microRNAs, histone modification and DNA methylation), mitochondrial dysfunction, immune responses and genetic factors (PNPLA3 and HSD17B13) contribute to NASH development and progression [23].

The gut microbiota, often referred to as the human “second genome,” plays a vital role in maintaining the digestive, metabolic, and protective functions of the gastrointestinal system. The existence of the gut-liver axis suggests a potential correlation between gut microbiota composition and liver diseases. Microbial communities and their metabolites can enter the liver via the portal vein, thereby influencing hepatic pathophysiological processes. Numerous studies have confirmed that dysbiosis of the gut microbiota contributes to the “multiple hits” involved in liver injury. It is recognized as a key driver in the onset and progression of NAFLD, playing a crucial role in its pathogenesis and accelerating its progression toward HCC [24]. Currently, the gut microbiota regulates the progression of NAFLD primarily through two mechanisms. First, the gut microbiota and its metabolites act as molecular mediators between the intestine and the liver. By altering intestinal metabolic outputs, the microbiota can influence host energy metabolism. Metabolites such as monosaccharides, short-chain fatty acids (SCFAs), bile acids (BAs), and trimethylamine (TMA) not only participate in the energy metabolism of intestinal epithelial cells and hepatocytes but also directly modulate systemic inflammatory responses and hepatic lipogenesis [24–26]. Second, the gut microbiota plays a key role in modulating immune functions of both the gut and liver. Under stimuli such as unhealthy lifestyles, the intestinal mucosal barrier becomes compromised, leading to increased gut permeability. Consequently, a large number of microbial metabolites, bacterial components, and pro-inflammatory molecules can translocate to the liver via the portal vein, exacerbating inflammation, oxidative stress, and lipid accumulation. These cumulative insults accelerate hepatic injury and fibrosis, thereby promoting NAFLD progression [24, 27].

Epigenetic modifications, as reversible and heritable changes in gene expression regulation, serve as a bridge between genetic susceptibility and environmental influences. These modifications are affected by lifestyle and dietary factors and play a significant role in the development and progression of nonalcoholic fatty liver disease (NAFLD). Among them, DNA methylation is the most extensively studied epigenetic mechanism in NAFLD. Studies have demonstrated that the methylation and gene expression of key enzymes involved in lipid and glucose metabolism exhibit disease-specific alterations in NAFLD [28]. Furthermore, dietary methyl donors can influence the methylation patterns of specific genes [29]. Histone modifications also impact NAFLD pathogenesis by modulating the transcriptional activity of genes involved in lipid metabolism, inflammation, and fibrosis. SIRT1, a key regulator of histone acetylation, has been shown to reduce hepatic lipid and triglyceride accumulation upon activation, thereby improving NAFLD outcomes [30]. In addition, microRNAs act as important mediators of various metabolic disorders. Several miRNAs, including miR-122, miR-27b, miR-33, miR-34a, and miR-223, have been identified as critical regulators of hepatic fatty acid metabolism and cholesterol homeostasis [31]. Among them, miR-122 is the most abundant miRNA in the liver and has been shown to suppress SIRT1 expression, thereby promoting lipogenesis and triglyceride secretion. It plays a pivotal role in maintaining hepatic homeostasis and lipid metabolism [32].

**Fig. 2:**
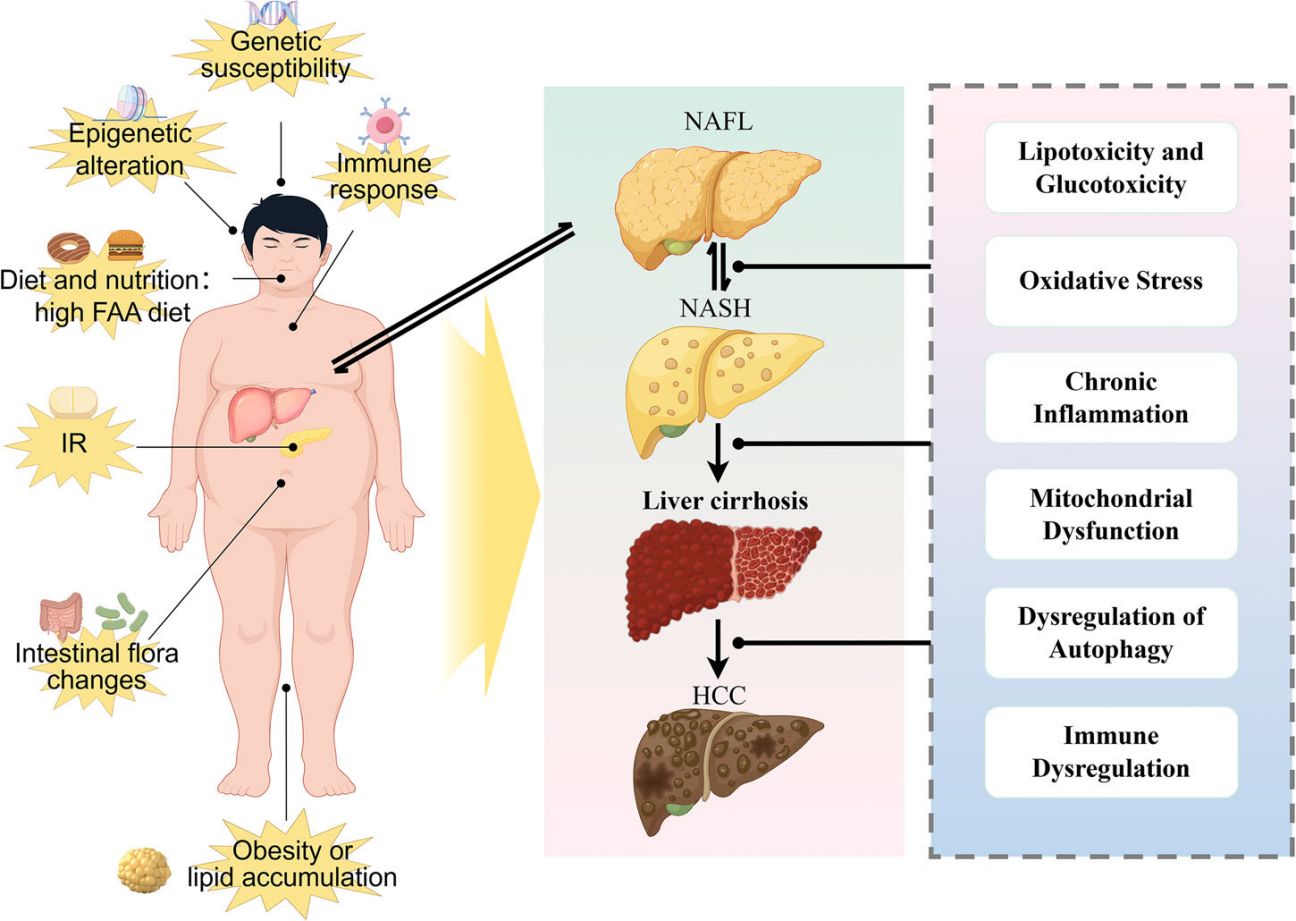
The etiology and pathogenic mechanisms of NAFLD/NASH and its promotion of HCC development.

Jorge Gutiérrez-Cuevas et al. summarized six key factors promoting the development of NASH-HCC: lipotoxicity and glucotoxicity, oxidative stress, chronic inflammation, mitochondrial dysfunction, autophagy dysregulation, and immune dysregulation [22]. The pathogenic mechanisms of NAFLD/NASH and their role in HCC development are presented in Fig. 2.

### Role of RCD in NASH-HCC progression and development

RCD mechanisms are essential for maintaining tissue homeostasis in a healthy liver. However, in chronic liver diseases such as NAFLD, which promote tissue fibrosis, cirrhosis, and HCC, it triggers adverse responses to cell death. In NAFLD, cell death is the ultimate consequence of oxidative stress and ER stress and serves as the driving force for disease progression and fibrosis [33]. RCD is a precisely orchestrated biological process controlled by specific molecules and signaling pathways. It is intrinsically linked to inflammatory liver diseases and is increasingly recognized as a critical factor in controlling the clinical outcomes of liver diseases [34]. Furthermore, emerging research has established significant associations between various RCD pathways and NAFLD [35]. The histological manifestations of NASH include hepatocyte ballooning, apoptosis, necroinflammation, and progressive fibrotic changes in the context of hepatic lipid accumulation. These features, which resemble the morphological characteristics of multiple RCD forms, indicate the involvement of various types of RCD in NASH [36]. Different RCD modes, including apoptosis, necroptosis, pyroptosis, and ferroptosis, play significant roles in NAFLD and the progression of NASH to HCC.

## Mechanisms of regulatory cell death

### Mechanism of apoptosis

**Fig. 3:**
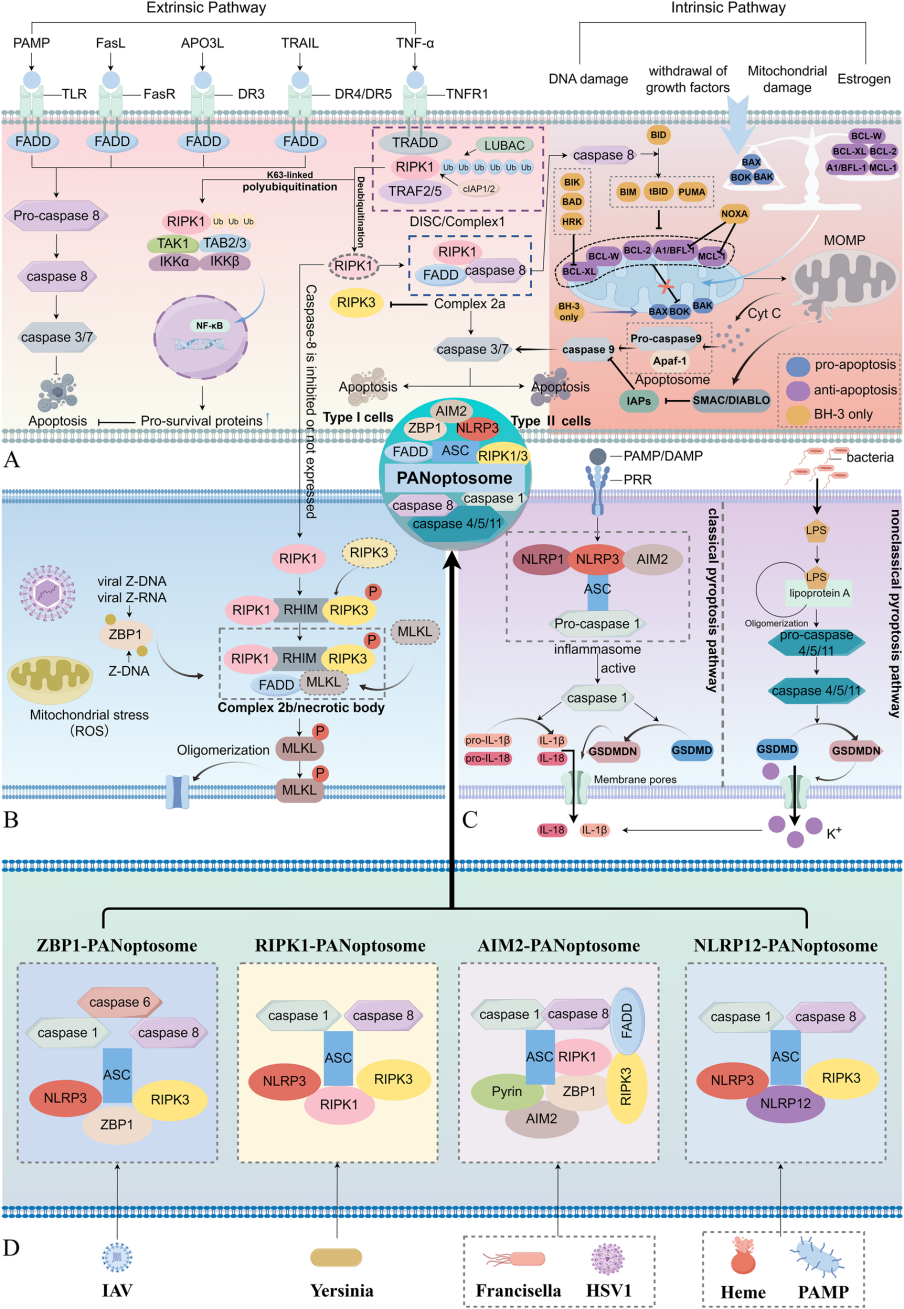
The mechanisms of apoptosis, pyroptosis, and necroptosis, as well as the formation of the PANoptosome. **A** The mechanisms of apoptosis, including extrinsic and intrinsic pathway. **B** The mechanisms of necroptosis **C** The mechanisms of pyroptosis. In the intrinsic pathway of apoptosis, BIM, PUMA, and tBID exhibit high affinity for all pro-survival proteins, and NOXA only binds to MCL-1 and A1/BFL-1, while BAD, BIK, and HRK primarily bind to BCL-XL, with occasional binding to BCL-2 and BCL-W. **D** Different sensors can recognize distinct stimuli and subsequently recruit key proteins from apoptosis, pyroptosis, and necroptosis pathways to collectively form a corresponding PANoptosome. This complex typically consists of sensors, adapter proteins, and catalytic effectors, functioning as a molecular scaffold. Ultimately, this multifunctional platform orchestrates PANoptosis, comprising apoptosis, necroptosis, and pyroptosis, leading to programmed cell death.

Apoptosis is a crucial intracellular mechanism for preserving homeostasis and regulating cell number. This programmed cell death process manifests through two distinct molecular pathways: extrinsic apoptosis, initiated by death receptor activation and intrinsic apoptosis, mediated by mitochondria. Both pathways ultimately lead to cell death by activating executioner caspases, specifically caspase-3 and caspase-7, resulting in protein degradation, nuclear fragmentation, and apoptotic cell death (Fig. 3A).

#### The intrinsic pathway of apoptosis

The mitochondrial-mediated apoptotic pathway, commonly referred to as intrinsic apoptosis, is characterized by mitochondrial outer membrane permeabilization (MOMP) [11]. This process can be activated by cellular changes, including DNA damage, mitochondrial damage, withdrawal of growth factors and estrogen signaling [17, 37]. However, anoikis, one of the intrinsic apoptotic pathways, can be induced by the loss of adhesion to the extracellular matrix or improper adhesion [38]. MOMP regulation is governed by the dynamic equilibrium between pro-apoptotic and anti-apoptotic members of the Bcl-2 protein family, which serves as a critical regulatory hub in apoptotic signaling [39]. The BCL-2 protein family can be functionally categorized into three subgroups: pro-apoptotic BH3-only proteins, pro-survival proteins, and pro-apoptotic effector proteins [40]. The pro-apoptotic effector proteins, including BAX, BAK, and BOK, can bind to the mitochondrial outer membrane during apoptosis, disrupting its integrity and promoting cell death. BAX and BAK are essential effectors of apoptosis with significant functional overlap, whereas BOK plays a supporting role [40]. The anti-apoptotic subgroup encompasses BCL-2, BCL-XL, BCL-W, MCL-1, and A1/BFL-1, with BCL-2, BCL-XL, and BCL-W exhibiting remarkable stability and prolonged half-lives, in contrast to the rapid proteasomal degradation of MCL-1 and A1/BFL-1 through ubiquitination [41, 42]. The BH3-only protein family includes BIM, tBID, BIK, PUMA, NOXA, BAD and HRK. BIM, PUMA, and tBID exhibit broad-spectrum binding capabilities to all anti-apoptotic proteins, thereby neutralizing their activities and initiating apoptosis. The actions of other BH3-only proteins are more selective; for example, NOXA only binds to MCL-1 and A1/BFL-1, whereas BAD, BIK, and HRK primarily bind to BCL-XL, with occasional binding to BCL-2 and BCL-W. Additionally, BIM, PUMA, and tBID can directly engage with BAX and BAK to initiate apoptosis [43, 44].

BID, a direct activator of apoptosis, undergoes transcriptional modification and cleavage to form tBID during apoptosis. Following mitochondrial translocation, tBID interacts with BAX and BAK, leading to MOMP formation and the subsequent release of cytochrome c (cyt c) and Smac/DIABLO from the mitochondria. Cyt c binds to APAF1 and pro-caspase 9, forming the apoptosome. Consequently, this activation cascade triggers the autocatalytic activation of executioner caspases 3 and 7, leading to cellular apoptosis [9, 45].

#### The extrinsic pathway of apoptosis

Extrinsic apoptosis, also known as the death receptor pathway, is initiated by the engagement of death receptors (DRs) located on the cytoplasmic membrane. This pathway is characterized by a death domain (DD), a specialized intracellular protein interaction domain. Examples of such DRs include FasR, TNFR1, LARD (DR3), TRAIL-R1 (DR4), TRAIL-R2 (DR5), and pattern recognition receptors (PRRs) like Toll-like receptors (TLRs) [46, 47]. When DRs bind to their corresponding ligands, a protein complex is formed, which induces downstream cascades. This leads to the subsequent activation of caspase 8, followed by caspase 3 and caspase 7, resulting in apoptosis. The two common pathways of receptor-ligand interaction are as follows: The FasL/FasR pathway mediates interactions between activated T cells and target cells. As a member of the TNF-R DR subfamily, FasR is ubiquitously expressed on various cell surfaces. Activated T cells express FasL, which binds to FasR on the target cell surface, recruiting FADD to the cytoplasmic side and activating caspase 8, thereby initiating the apoptosis cascade and triggering cell death. This mechanism protects immune cells from autoreactive lymphocyte attacks [48]. The TNF-α/TNFR1 pathway is driven by TNF-α secreted from T lymphocytes, activated macrophages and natural killer (NK) cells. When TNF-α binds to and activates TNFR1, it induces the rapid assembly of the death-inducing signaling complex, also known as complex 1, thereby regulating caspase 8 activity and its downstream signaling events. Complex I, a critical determinant of cellular fate, comprises multiple regulatory proteins including RIPK1, cIAP1, cIAP2, TRAF2, TRAF5, and the adapter TRADD. cIAP1 and cIAP2 produce polyubiquitin chains that help recruit other components of complex I, like the linear ubiquitin chain assembly complex. RIPK1, a key protein in complex I, plays a decisive role in TNF-α-mediated signaling outcomes, influencing whether the cell survives or undergoes apoptosis based on its ubiquitination status [49, 50]. K63 polyubiquitination of RIPK1 recruits TAK1, TAB2, and TAB3, thereby activating the NF-κB pathway and promoting the expression of anti-apoptotic genes that enhance cell survival. Conversely, deubiquitination of RIPK1 causes its release from complex I, where it associates with FADD and CASP8 to form complex IIa [51, 52]. After caspase 8 cleavage, the downstream activation of caspase 3 and caspase 7 results in apoptosis of lymphocytes and other type I cells [52]. In type II cells, a process consistent with the intrinsic pathway is required; BID is cleaved into tBID and translocated to the mitochondria, leading to MOMP and cell death [53]. These processes are mediated by RIPK1 and are a part of the RIPK1-dependent apoptosis (RDA) pathway. Moreover, TNF binding to TNFR1 can induce the RDA pathway [54].

### Mechanism of necroptosis

The discovery of necroptosis in 2005 challenged the notion that necrosis is a passive, unregulated form of cell death [55]. It was the first discovered PCD mechanism mediating necrosis. Necroptosis exhibits morphological features of necrotic cells while incorporating signaling pathways analogous to apoptosis. The release of intracellular content causes secondary inflammation and induces immune responses [13]. Like apoptosis, necroptosis can be initiated by the binding of DRs or PRRs to their respective ligands. Ligand-receptor binding facilitates the assembly of Complex I. During this process, deubiquitinated RIPK1 transitions to form Complex IIa, promoting RIPK1-dependent apoptosis. However, in the absence or inhibition of caspase-8, RIPK1 bypasses Complex IIa formation and instead recruits RIPK3, forming the RIPK1-RIPK3 complex via the RIP homology interaction motif. This complex phosphorylates RIPK3, which subsequently recruits the executive protein MLKL, forming Complex IIb, commonly referred to as necrosome [56, 57]. Phosphorylated MLKL translocates to the plasma membrane, where it forms oligomers and induces pore formation, causing necroptosis [56, 58]. In summary, the functional transition of RIPK1 can lead to three different outcomes: survival, necroptosis, and apoptosis. In addition to the aforementioned receptors, Z-DNA-binding protein 1 (ZBP1) can also initiate necroptosis by binding to Z-DNA or Z-RNA and interacting with RIPK3 through MLKL, or it can induce apoptosis via RIPK1, FADD, and caspase-8. Notably, Z-RNA is generated during viral infections, whereas Z-DNA is formed due to mitochondrial DNA (mtDNA) oxidation within the cytosol [59, 60]. The mechanism of necroptosis is illustrated in Fig. 3B.

### Mechanism of pyroptosis

Pyroptosis, also referred to as inflammatory necrosis, is a type of PCD associated with inflammatory responses [61]. It was first described by Cookson and Brennan in 2001 [62]. This process is critically mediated by the gasdermin protein family, with gasdermin D (GSDMD) being particularly significant. Proteins from the gasdermin family are cleaved at the N-terminal and C-terminal junction domains, releasing the activated N-terminal fragments. These activated N-terminal fragments bind to the plasma membrane and form pores, resulting in distinct cellular morphological alterations and eventual cell death [61, 63]. Two primary mechanisms of pyroptosis have been identified so far: canonical and the non-canonical pathways (Fig. 3C).

#### The classical pyroptosis pathway

The activation of this signaling cascade begins with the recognition of pathogen-associated molecular patterns (PAMPs) or damage-associated molecular patterns (DAMPs) by pattern recognition receptors (PRRs) [64, 65]. Proteins from the NOD-like receptor (NLR) family (for example, NLRP1 and NLRP3) and PYHIN family (for example, absent in melanoma-2 [AIM2]) recognize specific stimuli and recruit the adapter protein ASC. ASC facilitates inflammasome assembly by interacting with pro-caspase-1, leading to the activation of caspase-1 [66]. The activation of caspase-1 triggers the cleavage of GSDMD, releasing its N-terminal fragment, which translocates to the plasma membrane and forms pores, inducing pyroptosis. Additionally, caspase-1 processes pro-IL-1β and pro-IL-18 into their biologically active forms, which are subsequently released through the membrane pores, thereby initiating an inflammatory cascade [67]. Among the diverse mechanisms regulating inflammasome activation, particularly in the widely studied NLRP3 inflammasome, the interaction between NIMA-related kinase 7 (NEK7) and NLRP3 is a crucial step. This interaction is essential for inflammasome assembly and functional activation [68].

#### The nonclassical pyroptosis pathway

Unlike the classical pathway, the nonclassical pathway is triggered by cytosolic lipopolysaccharide (LPS) stimulation. Caspase-4, -5, and -11 directly recognize intracellular LPS and bind to its conserved structure, lipoprotein A, leading to oligomerization and formation of a large molecular complex. Similar to the classical pathway, caspase-4, -5, and -11 cleave GSDMD, and the cleaved N-terminal fragment of GSDMD relocates to the plasma membrane, forming pores and mediating pyroptosis [68, 69]. Furthermore, GSDMD-forming membrane pores enable potassium efflux, activating the NLRP3 inflammasome in the classical pathway and promoting the release of pro-inflammatory cytokines. It is noteworthy that, unlike apoptosis, pyroptosis occurs rapidly, is more pronounced, and involves the concurrent release of multiple pro-inflammatory mediators [68].

### Mechanism of PANoptosis

With in-depth exploration and discovery of the molecular mechanisms underlying different forms of RCD, apoptosis, necroptosis, and pyroptosis have been extensively elucidated, with complex dynamic molecular networks existing between them. The presence of multiple RCD pathways ensures that when one death pathway is suppressed, others can compensate and function to maintain tissue and organ homeostasis. This interdependence highlights that cell death processes are not entirely independent [70]. In 2019, Malireddi et al. proposed PANoptosis, a novel PCD mechanism that conceptually integrates the extensive interactions among pyroptosis, apoptosis, and necroptosis [71]. PANoptosis has recently garnered widespread attention as a new hotspot in RCD research. PANoptosis encompasses critical features of apoptosis, pyroptosis, and necroptosis. However, this cannot be completely explained by any RCD mechanism alone. PANoptosis involves the simultaneous participation of three key RCD modes, pyroptosis, apoptosis, and necroptosis, through various signaling pathways and molecular mechanisms [72]. The composition and function of the PANoptosis complex are central to its mechanism of action. In 2020, Christian et al. identified that molecules associated with pyroptosis, apoptosis, and necroptosis can assemble into a unified molecular complex, termed the PANoptosome [73]. The PANoptosome complex is a large molecular complex composed of various proteins. Samir et al. summarized the molecular components involved in PANoptosome formation [74]. Currently, the proteins constituting the PANoptosome can be classified into three main categories: (1) PAMP or DAMP sensors, including ZBP1, AIM2, and NLRP3; (2) adapter proteins, including ASC and FADD; (3) catalytic effectors, including RIPK1/3, caspase 1, caspase 4/5/11, and caspase 8 [74]. Based on the three types of molecules, PANoptosis integrates the cell death induction pathways of apoptosis, pyroptosis, and necroptosis. Specifically, PANoptosis summarizes the shared mechanism in which sensors detect upstream signals, adapter proteins serve as intermediaries to recruit and activate catalytic effectors, ultimately leading to cell death. According to current studies, PANoptosomes can be categorized into four subtypes based on their initiating sensors: ZBP1-PANoptosome, RIPK1-PANoptosome, AIM2-PANoptosome, and NLRP12-PANoptosome [75].

ZBP1 was initially identified as a Z-RNA sensor for influenza A virus (IAV), capable of activating the downstream NLRP3 inflammasome and inducing cell death. Notably, inhibition of a single regulated cell death (RCD) pathway is insufficient to prevent IAV-induced cell death, whereas deletion of ZBP1 effectively protects cells by blocking multiple RCD pathways triggered by IAV [76]. This phenomenon led to the discovery of the ZBP1-PANoptosome, the earliest described form of a PANoptosome. Upon sensing IAV infection, activated ZBP1 interacts with the CARD domain of ASC (CARD^ASC^) through one of its RHIM domains (RHIM2^ZBP1^) to recruit ASC. When RHIM2^ZBP1^ is occupied, the free RHIM1^ZBP1^ domain can recruit RIPK1 and/or RIPK3, eventually leading to the recruitment of effector molecules, completing the assembly of the PANoptosome and driving PANoptosis [74]. Additionally, CASP6, a key component of the PANoptosome, has been identified as a novel scaffold protein that enhances the interaction between RIPK3 and ZBP1, thereby facilitating PANoptosome assembly [77].

RIPK1, another sensor involved in PANoptosis, shares similarities with ZBP1 in its ability to sense Yersinia infection. It interacts with ASC, NLRP3, ZBP1, and CASP8, and is recruited to the PANoptosome to induce PANoptosis. However, unlike ZBP1, the loss of RIPK1 only eliminates Yersinia-induced apoptosis and pyroptosis, but it promotes ZBP1-mediated necroptosis, thus failing to completely prevent cell death [75, 78]. Moreover, the function of RIPK1 extends beyond its role as a sensor. Studies have demonstrated that the kinase-dead mutant of RIPK1 is still capable of inducing PANoptosis, confirming that RIPK1 can also act as an adapter or catalytic effector in certain contexts [79].

AIM2, a newly discovered sensor, is capable of recognizing pathogens, such as HSV-1 or Francisella tularensis, as well as cytosolic dsDNA released upon cell damage [80]. Subsequently, AIM2 can be delivered in an IFN-I-dependent manner, leading to the upregulation of its downstream proteins, pyrin and ZBP1. This cascade ultimately triggers the assembly of the AIM2-PANoptosome, initiating PANoptosis [81]. Interestingly, the assembly process of the AIM2-PANoptosome requires the participation of ZBP1.

NLRP12 is a newly discovered sensor, and its expression is regulated by TLR2 and TLR4 [75]. It has been shown that NLRP12 can recognize heme and PAMPs, thereby inducing the formation of the NLRP12-PANoptosome and driving cell death [82]. Current research on the NLRP12-PANoptosome remains limited. The precise mechanisms governing its assembly, along with the functional roles of its associated components, warrant further investigation.

Consequently, PANoptosome can be considered a molecular scaffold that houses key proteins capable of activating pyroptosis, apoptosis, and necroptosis, with these proteins playing different roles in the various types of RCD pathways (Fig. 3D). The upstream factors triggering PANoptosome assembly and the interactions and mechanisms of the proteins within it remain poorly understood. An in-depth analysis of all pathways regulated by the PANoptosome and the interactions of its components within the complex is crucial for future research. Targeting PANoptosis to regulate inflammatory cell death may exhibit therapeutic potential for treating inflammatory diseases and represents a promising target that requires further research to validate its feasibility.

### Ferroptosis

**Fig. 4:**
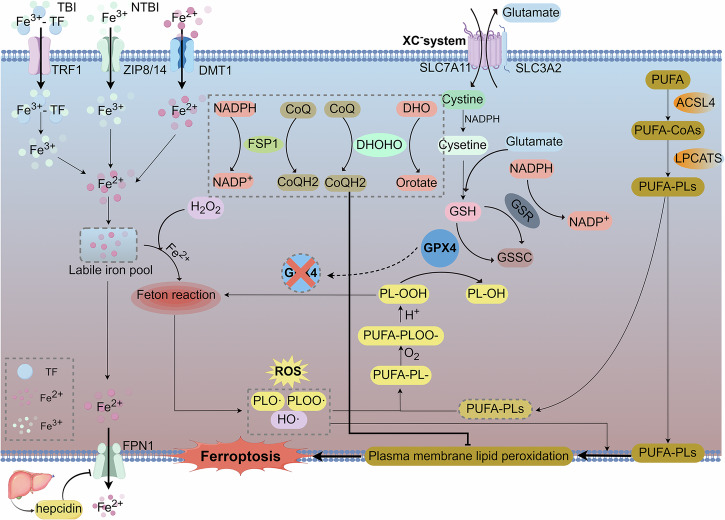
The mechanisms of ferroptosis.

Ferroptosis is a distinct form of RCD mediated by iron-dependent lipid peroxidation and differs from apoptosis, necroptosis, and pyroptosis. It was formally named by Stockwell in 2012 [83]. Unlike apoptosis, necroptosis, and pyroptosis, ferroptosis exhibits unique morphological features that do not involve the cellular mechanisms that govern apoptosis or necroptosis. Instead, it is regulated by specific signaling pathways. Ferroptosis is a metabolism-driven and regulated cell death mechanism initiated by iron-dependent lipid peroxidation, particularly of polyunsaturated fatty acyl chains within cellular lipids [83–85]. In this study, ferroptosis was delineated into the following key steps: (1) the formation of the labile iron pool (LIP) within the cell, (2) the generation and buildup of lipid peroxides, and (3) defects in the regulation and clearance of these peroxides, mediated by various mechanisms, including the XC-system/GSH/GPX4 axis (Fig. 4).

#### Formation of LIP within the cell

Extracellular iron exists as Fe^3+^ and is complexed with transferrin (TF), which is then recognized by the TF receptor 1 (TfR1) to facilitate its cellular uptake and subsequent reduction to Fe^2+^ (TF-bound iron). Under conditions of iron overload, extracellular Fe^3+^ can be directly transported into cells through zinc transporters 8/14 (ZIP8/14) as non-transferrin-bound iron. This process generates ROS, which can induce cell death and organ damage [86]. Within enterocytes, the divalent metal transporter 1 facilitates the uptake of extracellular Fe^2+^. The internalized Fe^2+^ is then stored in the LIP [86]. Hepatic regulation of systemic iron homeostasis is mediated by hepcidin, a peptide hormone synthesized by hepatocytes. Hepcidin binds to the iron transporter ferroportin-1, inducing its internalization and degradation, restricting iron efflux from hepatocytes and limiting intestinal iron absorption, thus playing a crucial role in maintaining iron homeostasis [87]. Fe^2+^ is essential for various enzymes involved in phospholipid peroxidation and ROS generation and is indispensable in the iron-dependent Fenton reaction, which is a key biochemical event in ferroptosis [88].

#### Formation and accumulation of lipid peroxides

Polyunsaturated fatty acid phospholipids (PUFA-PLs) are critical substrates for lipid peroxidation during ferroptosis. The biosynthesis of PUFAs is initiated through an enzymatic reaction mediated by acyl-CoA synthetase long-chain family member 4 (ACSL4), forming PUFA-CoAs, which are then catalyzed by lysophosphatidylcholine acyltransferase 3 (LPCAT3) to generate PUFA-PLs [89, 90]. The resulting PUFA-PLs accumulated in the cell membrane, thereby increasing their susceptibility to ferroptosis. When iron overload occurs within the cell, Fe^2+^ can produce ROS through the Fenton reaction. In the presence of esterases and iron, ROS react with PUFA-PLs in the cell membrane, triggering the production and accumulation of lipid peroxides, which subsequently promote cell death [89].

#### Regulation and clearance defects of peroxides

The XC^-^ System/GSH/GPX4 axis is the primary cellular mechanism that counteracts ferroptosis in mammals. The XC^-^ system, a cystine-glutamate antiporter, consists of the key subunit SLC7A11, which forms a heterodimer with SLC3A2. This transporter facilitates the influx of cystine into the cell, where it is reduced to cysteine, which then participates in the synthesis of glutathione (GSH) through a reaction with GSH [91]. Glutathione peroxidase 4 (GPX4), a pivotal enzyme in ferroptosis regulation, utilizes GSH to promote the reduction of lipid hydroperoxides (PL-OOH) to their corresponding lipid alcohols, promoting lipid peroxide clearance [90]. When GPX4 is inhibited or deficient, PL-OOHs persist longer, enabling them to undergo Fenton reactions with Fe^2+^ and Fe^3+^, producing highly reactive lipid radicals (PLO− and PLOO−). These free radicals then interact with PUFA-PLs, exacerbating PL-OOH accumulation and perpetuating a vicious cycle [88].

In addition to the GPX4-dependent mechanisms, several GPX4-independent pathways contribute to ferroptosis regulation, including the NAD(P)H/FSP1/CoQ10 axis, the GCH1/BH4/DHFR axis, squalene accumulation, and DHODH-mediated suppression of ferroptosis [88, 92]. Ferroptosis suppressor protein 1 (FSP1) functions as an NADH-quinone oxidoreductase, catalyzing the reduction of quinones or semiquinones to generate ubiquinol. Moreover, it facilitates the reduction of vitamin K to its antioxidant-active form, hydroquinone, which captures ROS, thereby mitigating oxidative stress and terminating lipid peroxidation [93, 94]. This mechanism protects against ferroptosis when GPX4 is absent or inhibited. Furthermore, Mao et al. identified DHODH as an independent factor in ferroptosis suppression in 2021 that was different from the canonical GPX4 pathway [92]. Several other mechanisms have also been implicated in ferroptosis resistance, although their precise molecular functions require further investigation [95, 96]. DHODH, FSP1, and GPX4 form a collaborative defense system that protects cells from ferroptotic death.

### Cuproptosis

**Fig. 5:**
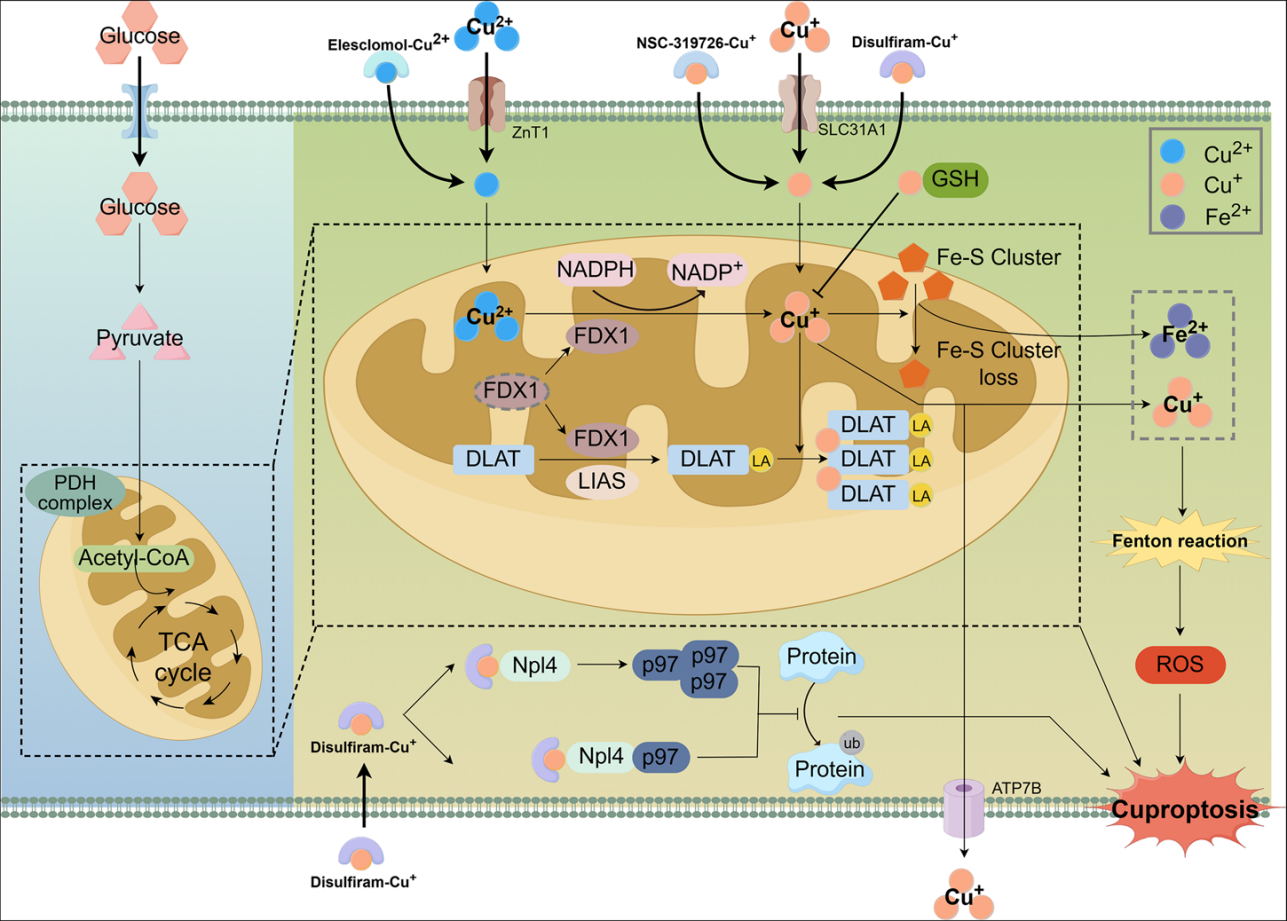
The mechanisms of cuproptosis.

Copper levels are typically maintained at low concentrations in mammalian cells. When intracellular copper ion (Cu^2+^) concentrations exceed the threshold that cellular homeostatic mechanisms can maintain, it induces cytotoxicity. Tsvetkov et al. proposed that cuproptosis is a distinct form of RCD, with a mechanism closely related to mitochondrial respiration and the lipoic acid (LA) metabolic pathway [97]. Cuproptosis exhibits morphological features similar to apoptosis; however, the underlying mechanisms differ (Fig. 5). Copper can enter cells through the copper transporter CTR1 (encoded by the SLC31A1 gene) and can be expelled via ATP7B. Additionally, copper can be transported across the plasma or mitochondrial membranes by copper ion carriers, including elesclomol (ES), NSC-319726, and disulfiram [98, 99]. Recently, zinc transporter 1 (ZnT1) was identified as a mediator of Cu^2+^ entry into cells and is essential for inducing cuproptosis [100]. During copper overload, the key proteins, ferredoxin 1 (FDX1) and LA synthase (LIAS), induce the acetylation of DLAT and the reduction of iron-sulfur (Fe-S) cluster proteins, which are central mechanisms in cuproptosis. FDX1 functions as a primary upstream modulator of DLAT acetylation, a critical element of the pyruvate dehydrogenase (PDH) complex, in cooperation with LIAS to facilitate DLAT acetylation [101]. Besides, FDX1 reduced Cu^2+^ to the more reactive and cytotoxic Cu+ species, thereby promoting the acetylation and oligomerization of DLAT. Moreover, the interaction between FDX1 and Cu^+^ destabilizes Fe-S clusters, leading to protein toxicity and cellular stress, ultimately triggering cuproptosis [97, 102]. The downstream pathways and direct mechanisms of acetylated DLAT oligomerization remain unclear.

In addition to the aforementioned mechanisms, copper overload-induced oxidative stress and inhibition of protein ubiquitination and degradation contribute to cuproptosis. Similar to ferroptosis, Cu^2+^ can generate a substantial amount of ROS via the Fenton reaction, leading to DNA damage, mitochondrial dysfunction, and lipid peroxidation, ultimately resulting in cell death [16, 103]. Furthermore, DSF-Cu can interact with Npl4, causing p97 aggregation, or can directly bind to p97, inhibiting its conformational changes and suppressing its ubiquitination function, resulting in cell death [104, 105]. However, GSH can act as a copper chelator during copper overload due to its thiol group, binding Cu^2+^ and reducing the occurrence of cuproptosis [106].

### Other modes of RCDs

In the current body of research literature, in addition to the six types of RCD mentioned above, other forms of RCD have also been described, including NETosis, parthanatos, lysosome-dependent cell death, alkaliptosis, oxeiptosis, and entotic cell death. However, a comprehensive literature review revealed insufficient reliable data regarding the involvement of these RCD pathways in NAFLD/NASH. Consequently, these mechanisms were excluded from the current review. Future investigations should prioritize elucidating the roles and implications of these cell death mechanisms in the context of NAFLD/NASH.

## Targeting RCD in NAFLD/NASH

### Targeting apoptotic pathways in NAFLD/NASH

Apoptosis is a pivotal mechanism in the development of NASH and associated HCC. In NASH, hepatocyte apoptosis significantly increases and is directly correlated with disease progression [107]. This process contributes to liver injury, fibrosis, and inflammation and promotes tumorigenesis [108].

#### Targeting the execution process of apoptosis

##### Mitochondrial dysfunction

The intrinsic and extrinsic apoptotic pathways ultimately converge in the mitochondria, making mitochondrial dysfunction a key event in apoptosis. In murine models of NAFLD, nicotine exposure downregulated the expression of CDGSH iron-sulfur domain 3 (CISD3), leading to impaired mitochondrial function. This aggravates oxidative stress and promotes apoptosis, highlighting the potential therapeutic effects of targeting CISD3 [109].

##### BCL-2

MOMP, a critical step in the intrinsic pathway, is modulated by the BCL-2 family. The acridone derivative A22 selectively binds and stabilizes the i-motif structure, a BCL-2 gene promoter, upregulating BCL-2 expression and reducing hepatocyte apoptosis in NAFLD/NASH models. This is the first attempt to treat NASH by targeting the i-motif structure of gene promoters using small molecules [110].

##### Mcl-1

Similarly, PNPT1 can degrade the mRNA of anti-apoptotic factor Mcl-1 under lipid-rich conditions and increase mitochondrial permeability. PNPT1 and Mcl-1 form a positive regulatory feedback loop that modulates hepatocyte apoptosis and maintains mitochondrial homeostasis [111].

##### BID

BID is an important neutralizing protein in the process of apoptosis. Eguchi et al. optimized the BID siRNA delivery system, enabling efficient accumulation in the liver, which effectively reduces BID levels and is accompanied by a decrease in mitochondrial BAX and BAK, thereby improving NASH-related liver fibrosis and alleviating the inflammatory response [112].

##### RIPK1

RIPK1, a critical factor determining the direction of apoptosis in the extrinsic pathway, has significant potential as a therapeutic target. SENP1 can deSUMOylate RIPK1 within the TNF-R1 signaling complex, thereby inhibiting RIPK1 activity and reducing cellular sensitivity to RIPK1 kinase-dependent apoptosis [113].

##### Caspases

Caspases are a group of crucial enzymes involved in apoptosis, with caspase 8 and caspase 3/7 serving as initiator and effector molecules, respectively, in the terminal stages of apoptotic pathways. Emricasan, a pan-caspase inhibitor, effectively inhibited apoptosis. In a 28-day Phase II clinical trial, emricasan administration significantly decreased the levels of caspase 3/7 and ALT levels, demonstrating a favorable safety profile and good tolerability [114]. In NASH mouse models, VX-166 attenuated the progression of hepatic fibrogenesis by suppressing hepatocyte apoptosis [114]. Emerging evidence suggests that caspase 2 activation plays a pivotal role in promoting the transition from NAFLD to NASH. Bosc et al. demonstrated that irreversible selective inhibition of caspase 2 using LJ2a and LJ3a (new peptidomimetics derived from the Val-Asp-Val-Ala-Asp [VDVAD] pentapeptide structure) effectively reduced adipocyte apoptosis and steatohepatitis [115].

#### Targeting the regulatory process of apoptosis

##### ASK1-p38/JNK pathway

Apoptosis is primarily induced through intracellular signaling pathways. In the context of NASH, a pivotal event in hepatocyte apoptosis involves the activation of apoptosis signal-regulating kinase 1 (ASK1), which induces dysregulation of glucose and lipid metabolism via downstream p38-JNK1 and JNK2 (JNK1/2) pathways, thereby promoting inflammation [116]. TNF-α-induced protein 3 is a key endogenous inhibitor of ASK1. It can inhibit ASK1 deubiquitination, thereby inactivating ASK1 and demonstrating potent anti-inflammatory and anti-apoptotic functions in NASH [116]. Similarly, He et al. recently developed a novel compound, 33c, which inhibited the upregulated protein expression of the ASK1-p38/JNK signaling pathway in TNF-α-treated HGC-27 cells and alleviated lipid accumulation in LO2 cells [117]. Selonsertib (GS-4997), a selective ASK1 inhibitor, has undergone phase 2 clinical trials for NASH treatment. However, it is associated with notable side effects (NCT02466516). The phosphorylation and activation of ASK1 are regulated by deubiquitination and dimerization. TIPE1 exerts a protective role in NASH by directly binding to ASK1, thereby inhibiting TRAF6-mediated polyubiquitination and ASK1 activation [118]. Moreover, direct inhibition of JNK activation using Glycogen Synthase Kinase-3 inhibitor IX and enzastaurin suppresses hepatocyte lipoapoptosis [119].

##### AMPM pathway

In NASH, the AMPK pathway is another important pathway regulating hepatocyte apoptosis. Zhao et al. published a study on the AMPK-caspase-6 axis in modulating liver injury in NASH, reporting that AMPK can phosphorylate the pro-apoptotic protein caspase-6, thereby inhibiting its activation and suppressing hepatocyte apoptosis [120]. Subsequent studies have confirmed the potential of AMPK as a promising molecular target for therapeutic intervention in NASH management. Ursodeoxycholic acid activates AMPK and influences the interaction of Bcl-2/Beclin-1 and Bcl-2/Bax complexes in NAFLD rats, thereby inhibiting apoptosis [121]. FTZ can also upregulate the expression levels of P-AMPK and BCL-2 while downregulating BAX, thereby improving steatosis and hepatocyte apoptosis to attenuate NASH [122]. The pathways regulating hepatocyte apoptosis in NASH are numerous and complex.

In addition to the aforementioned pathways, many other pathways are involved in hepatocyte apoptosis in NAFLD/NASH (Table 1).

**Table 1:** Key regulatory molecules of apoptosis in NAFLD/ANSH.

Regulatory axis	Effects on apoptosis	Target of action	Drug	Phase	Liver diseases	Brief mechanism	References
ASK1	Negative	ASK1	TNFAIP3	Animal experiment	NASH	Suppressor of ASK1 activation and deubiquitinase of ASK1 in hepatocytes	[129]
	Negative	ASK1	TIPE1	Animal experiment	NASH	directly binding to ASK1 and restraining its TRAF6-catalyzed polyubiquitination, inhibiting the activation of ASK1	[185]
	Negative	–	GS-4997	Clinical practice Phase III	NASH	Selective ASK1 Inhibitor	NCT02466516
ASK1-JNK1	Negative	ASK1	DUSP12	Animal experiment, Vitro experiment	NAFLD	directly interacting with ASK1 and inhibiting its activation	[244]
ASK1-p38/JNK	Negative	-	33c	Vitro experiment	NASH	Inhibiting the upregulated protein expression levels of the ASK1-p38/JNK signaling pathway and regulating apoptotic proteins.	[199]
IRE1α/TRAF2/JNK	Negative	–	PS-ALA	Animal experiment	NAFLD	inhibiting the IRE1α/TRAF2/JNK signaling pathway.	[245]
JNK/CHOP	Negative	–	Alpha-tocopherol	Animal experiment	NASH	lowering JNK/c-Jun/inflammation axis in addition to JNK/CHOP/ apoptosis signaling	[246]
AMPK	Negative	–	UDCA	Animal experiment	NAFLD	activating AMPK	[247]
	Negative	–	FTZ	Animal experiment	NASH	reducing the expression levels of Bax, cleaving caspase 3 and upregulating the expression of Bcl-2	[248]
	Negative	–	PS-ALA	Animal experiment, Vitro experiment	NAFLD	Improving ERS-triggered apoptosis by activating the AMPK	[245]
	Negative	–	GA	Vitro experiment	NASH	Suppressing apoptosis-related gene expression and caspase 3/7 activity	[249]
AMPK-caspase-6	Negative	–	–	Animal experiment	NASH	AMPK phosphorylates the pro-apoptotic caspase-6 protein to inhibit its activation	[250]
NF-κB	Negative	–	SOCS2	Animal experiment and Vitro experiment	NASH	Inhibiting inflammation and apoptosis via the NF-κB and inflammasome signaling pathway	[251]
KKβ/NF-κB	Positive	–	GA	Animal experiment, Vitro experiment	NAFLD- HCC	Attenuating the KKβ/NF-κB pathway	[171]
TLR-4/NF-κB	Negative	–	Scoparone	Animal experiment, Vitro experiment	NASH	blocking TLR-4/NF-κB signaling	[252]
NRF2/HO-1/NQO1	Negative	–	PIM1	Animal experiment, Vitro experiment	NAFLD	regulating the NRF2/HO-1/NQO1 signaling Pathway	[199]
HO-1	Negative	–	Heme oxygenase-1	Animal experiment, Vitro experiment	NASH	By suppression of ER stress and regulation of related gene and protein expression	[139]
CD1d-JAK2-STAT3	Negative	–	–	Animal experiment, Vitro experiment	NASH	anti-CD1d crosslinking leading to the recruitment and phosphorylation of JAK2	[111]
ATF6	Positive	ATF6	MiR-149	Animal experiment, Vitro experiment	NAFLD	Downregulate the expression of proteins related to the ATF6 signaling pathway	[253]
p53	Negative	–	Active vitamin D	Animal experiment	NAFLD	Suppression of the p53 pathway	[254]
ERK1/2/p65	Positive	–	IL-17A	Animal experiment	NASH	Via the activation of ERK1/2/p65 signaling pathway	[2]
STING-IRF3	Positive	–	–	Animal experiment	NAFLD	By disturbing glucose and lipid metabolism	[255]
TGF-β1/Smad3	Positive	p-Smad2/3	Ets-1	Animal experiment	NASH	p-Smad3 and enhancing the activity of TGF-β1/Smad3 signaling	[252]
caspase-3/PARP, ASK1/JNK	Negative	PRDX3	CAR	Animal experiment	NAFLD	maintaining mitochondrial membrane potential and reducing oxidative stress.	[256]

#### Application of antidiabetic drugs and androgens in targeting apoptosis

##### SGLT2 inhibitors

Selective sodium-glucose cotransporter 2 (SGLT2) inhibitors have gained significant attention as potential therapeutic agents for NAFLD and NASH. Numerous clinical studies have reported the beneficial impacts of SGLT2 inhibitors (SGLT2i) on NAFLD/NASH. Cagliazine, an SGLT2i, has been demonstrated to trigger apoptosis in HepG2 cells by activating caspase 3 cleavage, effectively suppressing the progression of NASH-HCC [123]. Furthermore, empirical evidence from a murine model of high-fat diet-induced NAFLD revealed that empagliflozin administration resulted in an elevated Bcl2/Bax ratio, suppression of Caspase-8 cleavage, and reduced hepatocyte apoptosis [124]. Although the mechanisms underlying the pro-apoptotic effects of cagliazine on HCC cells and the anti-apoptotic properties of empagliflozin in hepatocytes require further research, studies on the impact of the SGLTi family on HCC cells or hepatocyte apoptosis can be considered a promising direction for exploring targeted therapies for NASH-HCC. These results highlight that altering the direction of apoptosis regulation at different stages of disease progression may yield distinct therapeutic benefits.

##### Androgens

The androgen receptor (AR) signaling pathway plays a critical role in regulating hepatocyte growth and apoptosis, and its dysfunction may be implicated in the pathogenesis of liver diseases. Dihydrotestosterone (DHT), an active metabolite of the major circulating androgen testosterone, induces partial apoptosis in androgen-sensitive hepatocytes (such as SMMC-7721 and L02 cells) via the PKR/eIF2α/GADD153 cascade. Therefore, restoring the balance of AR signaling and inhibiting apoptosis in androgen-sensitive hepatocytes like L02 may offer potential therapeutic targets [125].

#### Targeting non-parenchymal cells in the liver

##### HSCs

In addition to focusing on hepatocytes, dysregulated lipid metabolism can promote NASH progression by activating hepatic stellate cells (HSCs) and inducing capillarization of liver sinusoidal endothelial cells (LSECs). Accordingly, targeting apoptosis in non-hepatocyte populations may offer novel therapeutic opportunities. Mishra et al. reported the development of a rationally designed protein, ProAgio, which induces apoptosis via the integrin αvβ3 signaling pathway. Integrin αvβ3 is overexpressed in activated HSCs, angiogenic endothelial cells, and capillarized LSECs. Through these mechanisms, ProAgio can reduce collagen deposition, reverse sinusoidal capillarization, and attenuate immune cell infiltration, thereby addressing the key pathological features of NASH progression [126]. Additionally, Rilpivirine inhibits STAT3 activation and improves fibrosis by selectively inducing HSC apoptosis via a STAT1-dependent pathway [127]. Moreover, in diet-induced murine models of NASH, CD8^+^ Trm cells facilitated the recruitment of HSCs via a CCR5-dependent mechanism, thereby sensitizing these cells to FasL-Fas-mediated apoptotic pathways [128].

##### CD4^+^ T cells

In NASH-HCC, the elimination of precancerous hepatocytes is critically dependent on CD4^+^ T cells. Suppression of peroxisome proliferator-activated receptor α (PPAR-α) attenuates CD4^+^ T cell apoptosis, thereby preserving immune homeostasis and contributing to the prevention of HCC development [129]. Mitochondrial sirtuin 4 inhibits PPARα activity, thereby suppressing lipid oxidation and AMP-activated protein kinase (AMPK) signaling pathways [130].

#### Role of traditional Chinese medicine in targeting apoptosis and other emerging perspectives

With the expansion of research fields and advancements in research technologies, new perspectives have emerged in targeting hepatocyte apoptosis for treating NAFLD/NASH-HCC. Mesenchymal stem cell-conditioned medium can alleviate hepatocyte inflammation and apoptosis by regulating SIRT1 [131]. In the context of gut microbiota, *Lactobacillus rhamnosus* HY7207 can modulate the expression of BAX and Bcl2, thereby reducing hepatocyte apoptosis and improving NAFLD in mice [132]. Telomeres and telomerase play crucial roles in the pathogenesis and progression of hepatic disorders. Telomerase promotes stem cell activation, inhibits apoptosis, mitigates oxidative stress, and enhances cellular vitality. PinX1 contributes to hepatocyte apoptosis and lipid accumulation by reducing telomere length and telomerase activity. The knockout of PinX1 attenuates the upregulation of cleaved caspase-3 expression, indicating its crucial role in regulating hepatocyte survival and lipid homeostasis [133].

Furthermore, apoptosis-induced progression of HCC in NASH may be closely associated with alterations in gene expression, which are modulated by changes in the expression and activity of histone-modifying enzymes responsible for acetylation and methylation processes. Although epigenetic-based pharmacological agents are currently undergoing clinical evaluation for HCC management, studies specifically focusing on NAFLD/NASH-HCC remain limited. Consequently, elucidating the molecular mechanisms of epigenetic modifications during apoptosis in NAFLD/NASH-HCC is imperative for developing novel biomarkers and targeted therapeutic interventions [134].

### Targeting necroptosis pathways in NAFLD/NASH

#### Targeting the execution process of necroptosis

The recognition of necroptosis as a unique PCD mechanism has led to substantial evidence linking it to NAFLD pathogenesis and its transition to NASH and HCC. This process significantly influences the degree of liver tissue damage. Activation of the RIPK1/RIPK3/MLKL signaling axis is a critical regulator of necroptosis.Consequently, the components of this pathway have become the primary therapeutic targets for researchers.

##### RIPK1

A RIPK1 inhibitor, necrostatin-1s, reversed necroptosis-mediated inflammation in Sod1KO mice after short-term treatment, thereby attenuating the progression of fibrosis and HCC [135]. Similarly, another RIPK1 inhibitor, RIPA-56, downregulated MLKL expression, thereby reducing liver injury, inflammation, and fibrosis in NASH [136].

##### RIPK3

RIPK3 inhibitor GSK-872 suppresses RIPK3 expression, mitigates hepatocyte necroptosis, and modulates the Nrf2/NFκB signaling axis to improve oxidative stress and inflammatory phenotypes [137]. Preston et al. discovered that epigenetic silencing of RIPK3 in hepatocytes prevented liver damage caused by MLKL-mediated necroptosis. Interestingly, RIPK3 was epigenetically silenced in human primary hepatocytes via methylation of its promoter region, thereby impairing their ability to undergo necroptosis [138]. This observation contrasts with the findings of Afonso et al., who reported elevated RIPK3 expression in liver biopsies from patients with NASH [139]. Consequently, further research into the methylation mechanisms governing RIPK3 expression may provide insights into its role in NASH pathogenesis and uncover potential therapeutic targets. Although targeting RIPK3 is a novel and promising therapeutic approach for NAFLD/NASH and HCC, it is crucial to consider that inhibiting necroptosis should not inadvertently activate other alternative cell death pathways in clinical practice. This is particularly important because RIPK3 deficiency can exacerbate inflammation, hepatocyte apoptosis, and early fibrosis, thereby promoting disease progression [140]. Furthermore, when using RIPK3 as a therapeutic target for NASH, the patient population must be carefully selected, as its inhibition may exacerbate insulin resistance and intolerance in obese individuals [141].

##### MILK

MLKL, the terminal effector of necroptosis, is another potential therapeutic target. In addition to inhibiting necroptosis signaling, MLKL deficiency protects against NAFLD by attenuating DNL and reducing the expression of chemokine ligands (CXC motif) [142, 143].

#### Targeting the regulatory process of necroptosis

In addition to direct inhibition of necroptosis, several other targets involved in necroptosis regulation have demonstrated therapeutic potential. Derlin-1 may accelerate NASH progression by exacerbating ER stress and necroptosis by modulating the ER-associated degradation pathway [144]. The transcription factor ATF3 facilitates RIPK3 expression, thereby shifting the predominant cell death pathway of hepatocytes from apoptosis to necroptosis during the exacerbation of hepatic steatosis. Consequently, inhibition of ATF3 could mitigate necroptosis [145]. Forkhead box protein O1 (FOXO1) is another key effector in the induction of necroptosis in NAFLD, while serpina3c suppresses necroptosis by downregulating the β-catenin/FOXO1/TLR4 signaling axis, thereby inhibiting NAFLD progression [146]. Moreover, in a rapid mouse model of liver inflammation, ELA significantly reduced RIPK3-mediated necroptosis in NASH by acting on PPAR-α and PPAR-δ [147]. Moreover, the polarity protein AF6 has been identified as a direct interactor with RIPK1’s intermediate domain, facilitating its ubiquitination status through modulation by the deubiquitinating enzyme USP21 and promoting hepatocyte necroptosis [148].

#### Targeting non-parenchymal cells in the liver

Within the hepatic microenvironment, necroptosis has been identified in non-hepatocyte populations, further influencing NASH pathogenesis. Sustained high-fat conditions downregulate UCP1 expression, with lower UCP1 levels driving necroptosis in NK cells, thereby compromising their ability to suppress the advancement of NASH-associated fibrosis [149]. In liver sinusoidal ECs, Mlkl knockout inhibits the activation of the TGF-β/Smad 2/3 pathway, thereby interrupting the pro-fibrotic crosstalk between ECs and HSCs, ultimately alleviating fibrosis in NASH [150]. Similarly, TTP reduces TNF-α expression in Kupffer cells, another type of non-parenchymal cell, thereby mitigating necroptosis in hepatocytes [151].

### Targeting the pyroptosis pathways in NAFLD/NASH

Previous studies have primarily focused on the fundamental mechanisms underlying NASH pathogenesis, including apoptosis and necrosis. However, pyroptosis has emerged as a focal point of interest due to its ability to compromise cellular membrane integrity and exacerbate NASH progression by releasing substantial amounts of intracellular inflammatory cytokines [152]. Numerous studies have reported significant elevations in pyroptosis-associated biomarkers in patients with NASH [153, 154]. Accordingly, the targeted regulation of hepatic pyroptosis provides a promising avenue for the prevention and therapeutic intervention of NASH.

#### Targeting the execution process of pyroptosis

##### NLRP3

Targeting the direct mediators of pyroptosis has been extensively investigated. NLRP3, a pivotal biomarker of NASH, drives pyroptosis in primary hepatocytes derived from murine and human sources upon activation, accompanied by the release of NLRP3 inflammasome-associated proteins. This process amplifies and perpetuates inflammasome-driven fibrosis, exacerbating disease progression [154]. Currently, NLRP3 inhibitors are used clinically to attenuate inflammation induced by hepatic steatosis. Compounds like CY-09, tranilast, and rabinosin reduce intracellular lipid droplet accumulation and ameliorate hepatic steatosis [155]. Other NLRP3 inhibitors, including MCC950 and rudosin, have demonstrated promise in preventing liver injury and fibrosis by suppressing pyroptosis and modulating anti-inflammatory effects [156]. Furthermore, vitamin D has demonstrated therapeutic effects in NAFLD mouse models, thereby inhibiting pyroptosis by suppressing NLRP3 inflammasome activation and reducing lipid accumulation. However, the specific pathways and mechanisms by which vitamin D inhibits NLRP3 remain to be elucidated [157].

##### AIM2

AIM2, another inflammasome implicated in pyroptosis, can be activated by mtDNA released from damaged mitochondria, thereby promoting hepatocyte pyroptosis and exacerbating NAFLD progression. Downregulation of the IRF1 gene inhibited mtDNA synthesis, attenuating AIM2 inflammasome activation and pyroptosis, providing a potential therapeutic target for NAFLD [158]. Notably, the specific NLRP3 inflammasome inhibitor MCC950 reduced the expression of AIM2 inflammasome-related genes in high-fat-treated hepatocytes in the same study, suggesting that the inhibitory effects of MCC950 may not be restricted to NLRP3 but could also extend to AIM2 [158].

##### GSDMD

GSDMD-mediated pyroptosis is commonly observed in patients with NASH, and the GSDMN domain has been proposed as a potential diagnostic biomarker for NASH. In GSDMD-knockout mice, the lipogenesis gene Srebp1c was downregulated, whereas PPARα and its downstream targets involved in lipid catabolism were upregulated. This resulted in significant amelioration of hepatic inflammation, steatosis, and fibrosis, highlighting the pivotal role of GSDMD-driven pyroptosis in NASH pathogenesis [153].

##### Caspase

Caspase-11, a “cutter” for GSDMD, plays a crucial role in NASH pathogenesis. In the livers of NASH mice, caspase-11 upregulation has been observed. Caspase-11-deficient mice exhibit suppressed activation of GSDMD and interleukin-1β, leading to reduced liver injury, fibrosis, and inflammation [159]. Additionally, VX-765 specifically inhibits caspase-1, significantly attenuating the pro-pyroptotic and pro-fibrotic effects of the p-STAT3/ANXA2 axis [160]. However, studies targeting other direct mediators of pyroptosis, including caspase-4 and caspase-5, in the context of NASH-HCC remain scarce, warranting further research into their therapeutic potential.

#### Targeting the regulatory process of pyroptosis

##### RNase

Targeting the indirect mediators of pyroptosis to treat NASH-HCC is worth considering. The RNase function of inositol-requiring enzyme 1 alpha (IRE1α) exhibits hepatocyte-specific activity, mediating NLRP3 inflammasome activation and cell death. The Bax inhibitor 1 and two small-molecule compounds STF-083010 and 4μ8c, inhibit IRE1α RNase activity, representing potential as therapeutic candidates for NASH management [161].

##### Transcription factor

Deficiency of the hepatocyte-specific transcription factor Nuclear Receptor Subfamily 5 Group A Member 2 (NR5A2) downregulates aldehyde dehydrogenase 1 family member B1 (ALDH1B1), resulting in increased ROS-induced NF-κB pathway activation, subsequently triggering pyroptosis. These molecular insights position ALDH1B1 as a critical therapeutic target for NASH. However, no ALDH1B1 activators have been identified to date [162]. Similarly, Caveolin-1, an important regulator of metabolic function, is involved in ROS scavenging. It inhibits NLRP3-dependent pyroptosis through the ROS/TXNIP/NLRP3 signaling cascade [163]. Another transcription factor, p-STAT3, interacts with the ANXA2 promoter region, thereby enhancing its transcriptional activity. ANXA2 expression is positively correlated with hepatocyte pyroptosis and fibrosis. Pharmacological inhibition of p-STAT3 markedly reduces both hepatocyte pyroptosis and fibrosis [160].

##### TLR4

TLR4 mediates NLRP3 inflammasome activation in NASH by binding to HMGB1. Molecular docking experiments have demonstrated that nicotinic acid interacts with TLR4, inhibiting the assembly of the NLRP3-ASC-Caspase-1 complex, suppressing pyroptosis, and alleviating NASH [164]. Furthermore, molecular docking studies have confirmed that raffinose targets Nrf2 and ameliorates pyroptosis in NAFLD by suppressing the TLR4-MyD88-NF-κB pathway [165].

##### NLRC4

During NAFLD progression, elevated TNF-α levels facilitate NLRC4 inflammasome activation, thereby triggering pyroptosis, exacerbating the inflammatory response, and promoting disease progression. Furthermore, silencing TNF-α in Hep G2 cells attenuated NLRC4 inflammasome activation [166]. A previous study revealed that NLRC4 inflammasome activation in NASH is independent of bacterial components. The results of this study revealed that DAG, produced by sphingomyelin synthase 1 (SMS1), can activate PKCδ and the NLRC4 inflammasome, leading to hepatocyte pyroptosis through the SMS1-PKCδ-NLRC4 signaling axis. Notably, SMS1 knockdown prevented NASH [167].

##### SIRT1

Emerging evidence suggests that pyroptosis inhibition represents a novel mechanism underlying the anti-inflammatory properties of SIRT1. The plant sterol ester of α-linolenic acid modulated the SIRT1 pathway, reducing NLRP3 and ASC expression, thereby preventing pyroptosis [168].

##### Autophagy

Autophagy, a self-protective cellular mechanism, plays a crucial role in pyroptosis. Taurine reduced As_2_O_3_-induced NASH through the autophagy-CTSB-NLRP3 inflammasome pathway [169].

##### Non-coding RNAs

The regulatory role of non-coding RNAs (ncRNAs) in pyroptosis is of significant interest in the context of NASH. CircSOD2 functions by competitively sponging miR-532-3p, which activates the TXNIP/NLRP3 inflammasome signaling pathway, thereby promoting pyroptosis during NAFLD progression [170]. Overexpression of long ncRNA (lncRNA) MIR22HG promotes autophagy through the miR-9-3p/IGF1 pathway, thereby inhibiting pyroptosis and fibrosis and attenuating NASH progression [171]. Similarly, lncRNA GAS5 acted as a sponge for miR-28a-5p, preventing it from targeting MARCH7. GAS5 inhibits NLRP3-mediated pyroptosis through MARCH7 suppression, thereby improving NAFLD. Consequently, the GAS5/miR-28a-5p/MARCH7/NLRP3 axis plays a crucial role in NAFLD progression [172].

#### Application of antidiabetic drugs in targeting pyroptosis

Recent studies have demonstrated that antidiabetic drugs may influence NASH progression by modulating pyroptosis signaling pathways. Taurine can regulate pyroptosis through autophagy, while liraglutide inhibits NLRP3 inflammasome-induced hepatocyte pyroptosis through mitochondrial autophagy [173]. Similarly, exenatide, another GLP-1 receptor agonist, reduced the expression of pyroptosis-related factors, leading to a reduction in fibrosis and inflammation [174]. Canagliflozin, an SGLT-2i, improves FGF21 activity, thereby inhibiting NLRP3-mediated pyroptosis through the FGF21-ERK1/2 signaling pathway [175]. Conversely, metformin promotes NAFLD progression in leptin-resistant individuals. Metformin, a widely used antidiabetic agent, activates AMPK and its downstream target cyt C oxidase, resulting in excessive activation of glucose catabolism-related genes. This dysregulation leads to energy depletion and triggers hepatocyte pyroptosis [176].

#### Targeting non-hepatocyte cell populations

##### HSCs

In addition to hepatocytes, we investigated pyroptotic mechanisms across diverse cell populations and their clinical relevance in NAFLD/NASH-HCC. When hepatocytes undergo pyroptosis upon activation of the NLRP3 inflammasome, they release inflammasome components that are subsequently engulfed by HSCs. This uptake leads to enhanced IL-1β secretion and increased expression of α-smooth muscle actin expression in HSCs. Notably, pretreatment of HSCs with the endocytosis inhibitor cytochalasin B can mitigate these effects, suggesting potential therapeutic applications for NASH management [154].

##### KCs

Antcin A, a small triterpenoid molecule, targets the NLRP3 inflammasome in Kupffer cells (KCs), inhibiting its assembly and activation. This subsequently suppresses pyroptosis in KCs, thereby alleviating the inflammatory response in NAFLD [177]. The multifunctional role of Caspase-11 extends beyond its canonical function in GSDMD cleavage-mediated pyroptosis. This protease plays a pivotal regulatory role in hepatic macrophages, including glycolysis, dual-fuel bioenergetics and oxidative phosphorylation. This metabolic regulation significantly promotes macrophage pyroptosis, facilitating the transition of NAFLD to NASH. Caspase-11 deficiency can reduce the effects of pyroptosis [178].

##### Intestinal macrophages

Furthermore, Poria cocos polysaccharides inhibit pyroptosis via PARP-1 modulation in intestinal macrophages, enhancing macrophage survival in high-fat environments, reducing gut vascular barrier disruption, and decreasing endotoxin translocation, thereby alleviating NASH progression [179].

### Targeting the PANoptosis pathways in NAFLD/NASH

PANoptosis, a newly identified form of RCD, has garnered significant attention recently. This cellular process is governed by upstream sensor proteins and complex signaling pathways that converge to form polymeric assemblies, which subsequently function as activation platforms for downstream effectors. These regulatory components are compelling therapeutic targets for potential intervention strategies.

#### Targeting the execution process of PANoptosis

Mitochondrial dysfunction is closely associated with the initiation and progression of PANoptosis in NAFLD. The hallmark features of this dysfunction include elevated mitochondrial ROS levels, mtDNA damage, impaired ATP synthesis, and significant changes in mitochondrial structure and morphology [180]. The traditional Chinese herbal formulation Si-Wu-Tang significantly attenuates mitochondrial damage and structural alterations. This effect is mediated by the reduction in NOXA transcription and protein levels, which inhibits VDAC oligomerization and prevents mitochondrial permeability transition pore (mPTP) channel opening, thereby suppressing lipid overload-induced mtDNA release. Consequently, this process hinders the binding of mtDNA to ZBP1 and subsequent activation of PANoptosis in hepatocytes. Si-Wu-Tang effectively reversed hepatocyte PANoptosis in both MCD-induced murine models and in vitro systems, demonstrating protective effects [181]. In the same study, Z-VAD-FMK, a PANoptosis inhibitor targeting the entire caspase family, demonstrated synergistic enhancement of the hepatoprotective effects of Si-Wu-Tang [181].

#### Targeting the regulatory process of PANoptosis

Similarly, FNDC4 not only improves TNF-α-induced mitochondrial dysfunction in hepatocytes by increasing mtDNA copy number and enhancing the expression of OXPHOS complex subunits I, II, III, and V, but also activates AMPKα to inhibit PANoptosis, thereby reducing hepatocyte death. This makes FNDC4 an attractive therapeutic target for preventing hepatocellular injury in patients with MASLD [182]. Additionally, post-translational modification of specific proteins may also influence the progression of MASLD. For example, an increased ratio of acylated to deacylated ghrelin can promote MASLD development by counteracting the compensatory inhibition of TNF-α-induced PANoptosis [183]. Recent investigations have demonstrated that liproxstatin-1, a potent ferroptosis inhibitor, exhibits regulatory effects on PANoptosis pathways, effectively mitigating hepatic steatosis and steatohepatitis progression in murine models of MAFLD. These experimental findings establish a molecular connection between ferroptosis and PANoptosis, highlighting the need for further research [184]. Caspase-8 is a key initiator of apoptosis. However, subsequent research has revealed its role in pyroptosis induction. Notably, the expression of enzyme-inactive caspase-8 (C362S) triggered necroptosis and pyroptosis, resulting in embryonic lethality and inflammatory tissue damage in mice [185]. These findings highlight the significant crosstalk between the components of PANoptosis. The modulation of caspase-8 enzymatic activity, whether through activation, inhibition, or encoding gene knockout, demonstrates intricate associations with the regulation of PANoptosis. Elucidating the precise molecular mechanisms of caspase-8 within PANoptosis in the context of NAFLD/NASH-HCC represents a critical research direction for advancing therapeutic strategies and mechanistic understanding.

#### Insights into hepatic PANoptosis regulation outside the NAFLD/NASH context

As a relatively novel concept, PANoptosis has been the focus of limited research in the context of NAFLD/NASH. However, several studies have investigated its suppression in the liver under non-NAFLD/NASH conditions. Baicalin has been shown to inhibit PANoptosis by reducing mitochondrial Z-DNA formation in macrophages and preventing the assembly of the ZBP1-PANoptosome, thereby alleviating hepatic injury [60]. Additionally, research by Shi et al. highlighted the pivotal role of reverse electron transport (RET) and mtDNA in the induction of PANoptosis, suggesting that anti-RET reagents that block mtDNA oxidation may represent a novel class of PANoptosis inhibitors. In their study, pretreatment with anti-RET agents such as 1-methoxy PMS (MPMS) or dimethyl fumarate (DMF) not only alleviated hemophagocytic lymphohistiocytosis (HLH) pathology but also significantly reduced PANoptotic features in the liver and kidneys [186]. ZBP1 is a key sensor of PANoptosis. Kanglexin, a novel anthraquinone derivative, has been found to promote PANoptosis in hepatocellular carcinoma cells by upregulating ZBP1 expression and preventing its degradation [187]. Although ZBP1 has been widely studied in various diseases due to its critical role in PANoptosis, no research to date has addressed its involvement in NAFLD/NASH. Therefore, investigating whether ZBP1-mediated inflammatory cell death contributes to the pathogenesis of NAFLD/NASH is of considerable interest [188].

#### Contributions of Emerging Technologies and Approaches to Targeting PANoptosis

Currently, few targets related to PANoptosis have been identified in the context of NAFLD/NASH. However, emerging technologies and methodologies may invigorate future target discovery. Bynigeri et al. identified protein phosphatase 6 (PP6) holoenzyme components as regulators of TAK1 inhibitor-induced PANoptosis by analyzing results from a cell death-based genome-wide CRISPR screen. Their findings suggest that PP6 may serve as a therapeutic target under inflammatory conditions [189]. Additionally, Wang et al. optimized an immunofluorescence-based technique that enables single-cell analysis of PANoptosome assembly. This approach provides a foundation for elucidating the molecular basis of PANoptosis and the highly dynamic, multiprotein PANoptosome complex, thereby advancing future research on PANoptosis-targeted therapies [190].

Emerging therapeutic approaches targeting PANoptosis regulatory mechanisms have demonstrated promising efficacy. Specifically, mitochondrial-targeted antioxidants, anti-inflammatory agents targeting death receptor pathways, and compounds modulating ER stress pathways exhibit therapeutic potential [191]. However, despite the presence of PANoptosis in both NAFLD and NASH, its role in NAFLD/NASH-HCC remains unclear. Given the regulatory influence of PANoptosis on various forms of RCD and its therapeutic applications in other malignancies, it is plausible that PANoptosis could be leveraged to uncover novel anti-inflammatory and antitumor therapies for NAFLD/NASH-HCC. Future investigations should focus on elucidating the complex regulatory networks of PANoptosis across diverse animal models. Identifying the key therapeutic targets within these pathways can lay the foundation for more effective clinical studies and therapeutic strategies.

### Targeting the ferroptosis pathways in NAFLD/NASH

Hepatic ferroptosis plays a critical role in triggering inflammation during steatohepatitis. As early as 2012, studies identified that iron accumulation due to metabolic dysfunction in patients with NAFLD can exacerbate NASH progression [192]. Moreover, in the early stages of NASH, necrosis occurs before apoptosis. Unlike necroptosis, ferroptosis inhibition almost entirely protects hepatocytes from necrotic death, thereby reducing immune cell infiltration and inflammatory responses [193]. Consequently, ferroptosis could be a potential therapeutic target for preventing the onset of NASH.

#### Targeting the execution process of ferroptosis

##### Lipid peroxides

Traditional therapeutic strategies targeting ferroptosis primarily focus on the direct mediators involved in the ferroptosis pathway. Lipid peroxides are integral to ferroptosis, and Trolox, a known ferroptosis inhibitor, inhibits lipid peroxidation, thereby significantly mitigating necrotic cell death, infiltration of immune cells, and subsequent inflammatory cytokine expression [193]. Similarly, liproxstatin-1, another potent ferroptosis inhibitor, significantly reduces hepatic triglyceride and cholesterol levels, as well as key biomarkers of lipid peroxidation, including 4-hydroxynonenal and malondialdehyde. It effectively prevented steatosis and NASH in mice with MAFLD [184]. Furthermore, in both of the aforementioned studies, DFP, an iron chelator, has been identified to exert protective effects by inhibiting ferroptosis.

##### ACSL4

ACSL4 is a key enzyme involved in lipid peroxidation during ferroptosis. ACSL4 knockout downregulated lipid peroxidation and inhibited ACSL4/p38 MAPK/GPX4 pathway-mediated ferroptosis, ultimately inhibiting NAFLD progression [194]. Moreover, using rosiglitazone or ACSL4 Small interfering RNA (siRNA) can suppress ACSL4 expression, downregulate the Mfn2/IRE1α pathway, and reduce the levels of 5-hydroxy eicosatetraenoic acid, thereby significantly alleviating ferroptosis and NASH [195].

##### GPX4

Sodium selenite, a known activator of GPX4, enhances GPX4 levels in the livers of mice, thereby alleviating the severity of NASH [196]. However, ferroptosis is promoted by the upregulation of a non-canonical GPX4 transcript variant, inducible-GPX4 (iGPX4), under NAFLD conditions. Targeting iGPX4 selectively presents a potential strategy for inhibiting ferroptosis in the treatment of NAFLD [197].

#### Targeting the regulatory process of ferroptosis

##### GSH/GPX4 axis

With the continuous exploration of ferroptosis mechanisms, numerous pathways regulating ferroptosis in NAFLD/NASH have been identified, providing more directions for targeting ferroptosis pathways. The GSH/GPX4 axis, one of the main biological pathways of ferroptosis, plays a critical role, with GPX4 serving as a central regulatory factor. TRIM59 is highly expressed in NAFLD tissues, where it interacts with GPX4 and promotes its ubiquitination, thereby accelerating fatty degeneration and ferroptosis in NAFLD [198]. Moreover, MARCH3 can promote the ubiquitination and degradation of GPX4, thereby regulating hepatocyte ferroptosis and lipid accumulation. Besides, the increased secretion of IL-6 by macrophages under chronic hypoxia upregulates MARCH3 expression in hepatocytes. This implies that targeting MARCH3 may exhibit therapeutic potential, particularly in patients with NAFLD and obstructive sleep apnea-hypopnea syndrome [199]. Consequently, these two proteins associated with GPX4 ubiquitination may serve as potential therapeutic targets for NAFLD. Conversely, Tβ4 and BMP4 overexpression in hepatocytes can upregulate GPX4 expression, thereby inhibiting ferroptosis, reducing hepatic oxidative stress and lipid peroxidation, and providing protection against NAFLD/NASH [200, 201]. Furthermore, the expression of the mitochondrial calcium uniporter (MCU) is upregulated in PFOS-induced NASH. MCU not only suppresses GPX4 expression through the mtROS-NRF2 signaling axis but also promotes autophagy-dependent ferroptosis by inducing mitochondrial dysfunction, leading to elevated cytosolic iron levels and facilitating the MCU-ACSL4 interaction, which further enhances ACSL4 activity. The siRNA-targeting MCU can systematically reverse these alterations [202].

##### Transcription factor

ATF4 is a transcription factor, and spermidine can activate it to upregulate the SLC7A11/GCLM/GPX4 signaling pathway, inhibiting ferroptosis and improving cellular health, thereby alleviating NAFLD [203]. Additionally, it can suppress the occurrence of NASH-HCC [204]. During iron overload, nuclear translocation of the transcription factor EB enhances the autophagic degradation of ferritin and the uptake of lipids, thereby contributing to ferroptosis and aggravating the pathological progression of NASH [205].

##### Epigenetic and protein modifications

The regulatory roles of epigenetic and protein modifications in ferroptosis cannot be ignored. Arbutin can modulate the activity of the demethylase fat mass and obesity-associated protein, thereby inhibiting its role in regulating SLC7A11 methylation. This inhibition helps suppress ferroptosis, thereby alleviating NAFLD progression [206]. In patients with NASH, GCN5L1 upregulation facilitates CypD acetylation, enhancing its interaction with ATP5B. This interaction triggers the opening of the mPTP, leading to the leakage of ROS from the mitochondria into the cytoplasm. The elevated ROS levels promote hepatocyte ferroptosis further and contribute to HMGB1 accumulation in the microenvironment. This process recruits neutrophils and induces the formation of neutrophil extracellular traps (NETs). NET depletion ameliorates the progression of GCN5L1-induced NASH [207].

##### Melatonin

Melatonin has emerged as a key regulator of ferroptosis, primarily due to its indirect stimulation of antioxidant enzymes that modulate the cellular redox balance. In NAFLD, melatonin exerts protective effects by restoring the oxidative balance within cells, suppressing lipogenesis and lipid peroxidation, and modulating the Nrf2/HO-1 and GPX4/SLC7A11 signaling pathways to counteract ferroptosis. Conversely, it can also alleviate ER stress and inhibit ferroptosis via the MT2/cAMP/PKA/IRE1 pathway [208, 209].

##### Unsaturated fatty acids

In terms of lipid peroxidation, the involvement of unsaturated fatty acids in ferroptosis provides a unique and crucial perspective. Molecular docking studies have demonstrated that palmitoleic acid can bind to ACSL4, leading to decreased ACSL4 levels and increased expression of GPX4 and SLC7A11. This modulation suppresses ferroptosis in NAFLD, alleviates oxidative stress, and reduces hepatic iron levels [210]. Moreover, DHA-enriched diets alleviate NAFLD-associated characteristics and ferroptosis-induced hepatic injury in zebrafish; although the exact mechanisms remain poorly understood [211].

In addition to the aforementioned regulatory pathways, recent investigations have identified several other pathways that influence ferroptosis. In a zebrafish NAFLD model, IGFBP7 mediated ferritinophagy through nuclear receptor coactivator 4, thereby regulating ferroptosis and promoting NAFLD progression [212]. Furthermore, during ferroptosis, mitochondrial lipid peroxides trigger the hyperoxidation of PRDX3, prompting its translocation from the mitochondria to the plasma membrane, where it inhibits cystine uptake, ultimately leading to ferroptotic cell death [213]. Therefore, inhibition of IGFBP7 and PRDX3 represents a potential novel therapeutic target. Conversely, ZEA downregulates p53 expression in FFA-treated HepG2 cells, reducing ROS production and iron accumulation, thereby inhibiting ferroptosis [214]. Additionally, Enoyl-CoA hydratase 1 (ECH1) knockdown exacerbates NASH progression, while ferroptosis inhibition can reverse this effect [215]. Similar to ZEA, ECH1 inhibition may confer protection by blocking ferroptosis in NASH.

#### Application of antidiabetic drugs in targeting ferroptosis

Ferroptosis is closely associated with metabolic disorders. Antidiabetic agents, key components of diabetes management, regulate ferroptosis by modulating metabolic pathways. As previously described, rosiglitazone inhibits ACSL4 expression, thereby attenuating ferroptosis [195]. Similarly, metformin regulates the xCT/GPX4/ACSL4 axis, thereby mitigating ferroptosis in hepatocytes in NAFLD [216]. Moreover, liraglutide activates AMPK, leading to ACC phosphorylation and the subsequent suppression of ferroptosis [217]. The modulatory effects of these agents on ferroptosis have predominantly been observed in NAFLD associated with T2DM, demonstrating a pivotal role of glucose metabolism in the interplay between ferroptosis and NAFLD progression.

#### Regulation of ferroptosis through targeting microbiota and non-hepatocyte cells

##### Oral and gut microbiota

The oral and gut microbiota,and the interactions between various cell types and hepatocytes, play a key role in modulating ferroptosis in NAFLD. *Porphyromonas gingivalis* induces hepatocyte ferroptosis and inflammatory responses by activating the NF-κB signaling pathway, thereby aggravating liver injury [218]. Furthermore, oral administration of *P. gingivalis* disrupts microbial metabolism, resulting in a Th17/Treg imbalance that triggers hepatocyte ferroptosis and accelerates NAFLD progression [219]. These observations highlight the significant influence of periodontal health on the modulation of ferroptosis in NAFLD. When administered orally, Urolithin C mitigates the detrimental effects of NAFLD by modulating the AMPK-ferroptosis axis, preserving intestinal barrier integrity and preventing gut dysbiosis. This mechanism has only been observed in vivo studies and not in vitro studies, highlighting the essential role of the gut-liver axis in regulating ferroptosis [220].

##### Hepatic macrophages and monocytes

The crosstalk between other cell types and hepatocytes significantly affects ferroptosis in NAFLD. In hepatic macrophages and monocytes, EFHD2, a scaffold protein, was upregulated in both patients with NASH and murine models. EFHD2 facilitates IFN-γ signaling by regulating the YWHAZ/14-3-3ζ-dependent membrane translocation of IFNγR2, thereby driving ferroptosis in macrophages and monocytes. This process further influences adjacent hepatocytes through intercellular communication. Fu et al. designed a stapled peptide targeting EFHD2, which inhibited ferroptosis and alleviated fibrosis progression in NASH [221]. Moreover, this study implies that scaffold proteins could be druggable targets, thereby broadening the scope and feasibility of target screening.

##### Red blood cells

In terms of red blood cells, an increase in ectopic phagocytosis can exacerbate iron overload, while platelets can upregulate red blood cell phagocytosis in NASH livers, promoting cellular ferroptosis. Conversely, cilostazol can inhibit this effect and mitigate iron overload-mediated ferroptosis in NASH models [222].

#### Applications of emerging technologies and approaches in targeting ferroptosis

The emergence of new technologies and research methods has improved the precision and efficiency of such studies. Yu et al. developed a liver-targeted drug nanodelivery system by combining 18-β-glycyrrhetinic-acid-chitosan oligosaccharide-N-acetylcysteine polymer (GCNp) with curcumin (Cur) and formulated a novel drug, GCNp-Cur NPs. This formulation can inhibit hepatocyte ferroptosis via multiple molecular pathways, thereby mitigating NAFLD progression. The findings of this study offer valuable insights for the future development of therapeutic agents targeting hepatocyte ferroptosis, potentially improving targeting accuracy and reducing systemic side effects [223].

### Targeting the cuproptosis pathways in NAFLD/NASH

Targeted modulation of ferroptosis has demonstrated considerable efficacy in the therapeutic research of NASH-HCC. Copper, another important metal ion, significantly affects cell death and disease progression through its metabolic regulation. The liver is central to copper homeostasis, and disruptions in copper metabolism can induce mitochondrial dysfunction, subsequently triggering hepatocyte death and inflammation [224]. Cuproptosis is recognized as a pivotal factor in NAFLD pathogenesis, potentially driving the transition from NAFLD to NASH, cirrhosis, and ultimately HCC via different mechanisms, including oxidative stress, insulin resistance and inflammation [106]. These findings underscore the therapeutic promise of cuproptosis-targeted approaches in arresting NAFLD/NASH disease progression.

#### Targeting cuproptosis in the context of NAFLD/NASH

Compared to other RCD forms, cuproptosis is a relatively recent concept with limited research in the context of NAFLD/NASH-HCC. Most studies have focused on the bioinformatic analysis of public datasets. Ouyang et al. performed bioinformatics analysis using multiple machine learning methods on the GEO database and identified three pivotal cuproptosis-related genes (CRGs): NFE2L2, DLD and POLD1. The ceRNA network demonstrated intricate molecular interactions among these genes, and their transcription levels were validated in mouse NAFLD samples [225]. Similarly, Liu et al. identified three CRGs (ENO3, SLC16A1 and LEPR), and Li et al. identified two CRGs (DLD and DLAT) closely associated with NAFLD [226, 227]. Additionally, Wu et al. discovered that PDHB and DLD are closely related to cuproptosis and affect NAFLD prognosis, further elucidating the molecular mechanisms of copper-related death in NAFLD [228]. These findings were verified using clinical samples and animal models. Extensive research has established the pivotal involvement of cuproptosis in NAFLD development and progression, providing a solid foundation for further investigation of the molecular mechanisms of cuproptosis in NAFLD and the identification of potential therapeutic targets. FDX1, a key protein involved in cuproptosis, was identified as a critical CRG in both NASH and its malignant transformation to HCC in an integrative analysis of transcriptome and single-cell sequencing datasets. Its expression was observed in both NASH animal models and human patients, suggesting that targeting FDX1 could be a potential strategy to prevent the progression of NASH to HCC [229]. Moreover, CTR1 is a critical gateway that regulates copper influx into the cells. Endogenous copper can stimulate MYC expression, which subsequently upregulates CTR1 expression through the Cu/MYC/CTR1 axis, thereby enhancing copper influx into cells. Consequently, reducing the transcription of CTR1 has emerged as a promising therapeutic approach for managing NAFLD/NASH-HCC progression [230]. LIAS has also been identified as a key regulator of cuproptosis. In an obese mouse model, Xu et al. demonstrated that overexpression of the LIAS gene effectively reduces oxidative stress and improves mitochondrial function in the liver, thereby alleviating NAFLD/NASH [231].

#### Insights into hepatic cuproptosis regulation outside the NAFLD/NASH Context

Although studies on cuproptosis in the context of NAFLD/NASH remain limited, research in other liver disease models provides valuable insights for future exploration. Luo et al. employed a kinase inhibitor library and identified merestinib (MTB) as a potent inhibitor of cuproptosis. MTB was shown to bind directly to NRF2, reducing oxidative stress and promoting copper exocytosis and transport, thereby enhancing copper homeostasis and alleviating cuproptosis-induced acute liver injury in mice [232]. Interestingly, other studies have indicated that cuproptosis may accelerate the progression of MAFLD through mechanisms such as disruption of the tricarboxylic acid (TCA) cycle, activation of autophagy, and engagement of the NRF2 pathway [233]. In addition, exogenous spermidine administration has been reported to reduce hepatic accumulation of ROS, MDA, and Cu2+, thereby attenuating cuproptosis, oxidative damage, and inflammatory responses, offering a protective effect on the liver [234].

Beyond hepatocytes, the regulation of cuproptosis in HSCs may represent another promising therapeutic direction. Diallyl trisulfides (DATs), a garlic-derived compound, have been shown to target Ras-related protein Rab-18 (RAB18) and induce its phase separation. Moreover, DATs promote the formation of mitochondria-associated membrane structures(MAMs), further accelerating RAB18 phase separation. Through the RAB18/CPT1A/DLD pathway, RAB18 can induce HSC cuproptosis. As such, DATs selectively protect hepatocytes while inducing HSC cuproptosis, thereby exerting antifibrotic effects [235].

Currently, the majority of studies on cuproptosis have focused on HCC. Li et al. demonstrated that maternal embryonic leucine zipper kinase (MELK) promotes the expression of DLAT via the PI3K/mTOR signaling pathway, thereby enhancing mitochondrial respiration, reducing excessive intracellular ROS, alleviating oxidative stress and mitochondrial damage, and ultimately facilitating HCC progression [236]. In addition, lipoyltransferase 1 (LIPT1), a gene associated with cuproptosis, has been shown to be significantly correlated with dysregulated fatty acid metabolism in HCC patients and plays a critical role in lipid metabolic reprogramming. Silencing LIPT1 suppresses the expression of PPARγ and inhibits the AKT/GSK-3β/β-catenin signaling pathway, resulting in a marked reduction in intracellular lipid accumulation [237]. Therefore, these cuproptosis-promoting targets identified in HCC may also play a regulatory role during the NAFLD/NASH stage.

In the context of HCC, epigenetic modifications also show potential in regulating cuproptosis. Using bioinformatics approaches, Zhu et al. identified cg05706061 in the promoter region of the SLC31A2 gene, which is associated with cuproptosis. They found that the methylation level of cg05706061 was significantly positively correlated with the expression of SLC31A2, with lower DNA methylation levels of SLC31A2 associated with reduced cuproptosis in HCC [238]. Inhibition of SLC31A2 methylation may potentially reduce cuproptosis in the context of NAFLD/NASH.

Cuproptosis, a “new generation” in the field of RCD research, has gradually demonstrated its unique potential. However, the precise mechanisms underlying cuproptosis remain poorly understood. Future studies on the mechanisms of cuproptosis in NAFLD/NASH-HCC are crucial for identifying new regulatory pathways and their interaction targets. This is an indispensable step in unlocking the therapeutic potential of cuproptosis in NAFLD/NASH-HCC.

## Drugs targeting RCDs to treat NAFLD/NASH

RCD plays a crucial role in the progression of metabolic liver diseases to hepatitis and cancer. The regulatory factors of different RCD pathways remain highly attractive therapeutic targets in the context of related liver diseases. Therapeutic drugs with the potential to target RCD for treating NAFLD/NASH-HCC, as well as the key targets under investigation, are summarized in this review (Table 2). The classification of these drugs demonstrates that both natural compounds and synthetic agents can act directly or indirectly on one or more RCD pathways to exert anti-inflammatory, antitumor, and antifibrotic effects, thereby achieving the objective of targeted therapy for NAFLD/NASH-HCC. Moreover, treating the underlying diseases or complications associated with NAFLD/NASH-HCC can facilitate NAFLD/NASH-HCC prevention. Currently, the therapeutic targets of NAFLD/NASH drugs in clinical trials primarily focus on GLP-1R, SGLT, PPAR, FXR, HSD17B13, TLR, AMPK, THR-β, ACC, and the gut microbiota, among others. Additionally, dietary and lifestyle interventions are crucial components of clinical trials for treating NAFLD/NASH. As of May 11, 2025, a search of the ClinicalTrials.gov database using keywords related to NAFLD/MAFLD and NASH returned 240 studies. However, none of these trials involved drugs specifically targeting RCD for NAFLD/NASH treatment. Furthermore, to date, only Rezdiffra has been approved by the FDA for the treatment of NASH [239]. In Table 3, we summarize the drugs currently in clinical trials that have potential to target RCD for treating NAFLD/NASH.

**Table 2:** The therapeutic drugs or key targets under investigation with the potential to target RCD for treating NAFLD/NASH.

Function chennel	Drug	Attribute	Target	Biological effect	Disease	Phase	References
Apoptosis	TNFAIP3	A zinc finger protein and a ubiquitin-editing enzyme	ASK1	Suppressor of ASK1 activation and deubiquitinase of ASK1 in hepatocytes	NASH	Animal experiment	[116]
Apoptosis	TIPE1	An immune and tumor regulatory factor	ASK1	Directly binding to ASK1 and inhibiting the activation of ASK1	NASH	Animal experiment	[118]
Apoptosis	DUSP12	A member of the dual-specificity phosphatase (DUSP) family	ASK1	Directly interacting with ASK1 and inhibiting its activation	NAFLD	Animal experiment, Vitro experiment	[257]
Apoptosis	GSK-3 inhibitor IX, enzastaurin	Inhibitor of GSK-3	GSK-3	Inhibiting activation of JNK	NAFLD	Vitro experiment	[119]
Apoptosis	AMPK	AMP-activated protein kinase	caspase-6	Phosphorylating the pro-apoptotic caspase-6 protein to inhibit its activation	NASH	Animal experiment	[120]
Apoptosis	UDCA	A secondary bile acid	AMPK	By activating AMPK, UDCA influences the interactions of the Bcl-2/Beclin-1 and Bcl-2/Bax complexes.	NAFLD	Animal experiment	[121]
Apoptosis	PIM1	A pro-viral integration site for Moloney murine leukemia virus 1	NRF2/HO-1/NQO1	By regulating the NRF2/HO-1/NQO1 signaling Pathway	NAFLD	Animal experiment, Vitro experiment	[258]
Apoptosis	CAR	A kind of diterpenoid with antioxidant	PRDX3	Maintaining mitochondrial membrane potential and reducing oxidative stress.	NAFLD	Animal experiment	[256]
Apoptosis	A22	An acridone derivative	i-motif	By selective binding to and stabilizing BCL-2 gene promoter i-motif.	NAFLD/NASH	Animal experiment, Vitro experiment	[110]
Apoptosis	PNPT1	An exoribonuclease	Mcl-1 mRNA	The KH and S1 domains of PNPT1 bind to and degrade Mcl-1 mRNA	MAFLD	Animal experiment, Vitro experiment	[111]
Apoptosis	SENP1	A SUMO-specific protease	RIPK1	Removing SUMO from RIPK1 in the TNF-R1 signaling complex (TNF-RSC)	NASH	Animal experiment	[113]
Apoptosis	Emricasan	A pan-caspase inhibitor	caspase	Inhibition of Hepatocyte Apoptosis	NASH	Animal experiment	[114]
Apoptosis	VX-166	A pan-caspase inhibitor	caspase 3/7	Inhibition of Hepatocyte Apoptosis	NASH	Animal experiment	[114]
Apoptosis	LJ2a, LJ3a	Inhibitor of caspase 2	Caspase 2	Inhibiting activation of caspase	NASH	Vitro experiment	[115]
Apoptosis	Cagliazine	A selective sodium-glucose cotransporter 2 (SGLT2) inhibitor	SGLT2	By directly inhibiting SGLT2 in tumor cells	NASH	Animal experiment, Vitro experiment	[123]
Apoptosis	Empagliflozin	A SGLT-2 inhibitor	SGLT2	Increasing the Bcl-2/Bax ratio	NAFLD/NASH	Animal experiment	[124]
Apoptosis	ProAgio	A rationally designed protein	αvβ3	By inducing integrin αvβ3-mediated cell apoptosis	NASH/AH	Animal experiment	[126]
Apoptosis	Rilpivirine	A widely used anti-HIV drug	STAT1	Through selective STAT1-dependent induction of apoptosis in HSC	Liver fibrosis	Animal experiment, Vitro experiment	[127]
Apoptosis	perhexiline	A CPT inhibitor	CPT	Decreasing apoptosis of intrahepatic CD4 + T cells	NASH-HCC	Animal experiment, Vitro experiment	[129]
Apoptosis	PinX1	A telomerase inhibitor	telomerase	By decreasing telomere length and telomerase activity	NAFLD	Animal experiment	[133]
Necroptosis	RIPA-56	A highly specific inhibitor of RIPK1	RIPK1	Decreasing MLKL activation	NAFLD	Animal experiment	[136]
Necroptosis	GSK-872	A specific inhibitor of RIPK3	RIPK3	Modulating Nrf2/NFκB signaling pathway	NAFLD	Animal experiment, Vitro experiment	[137]
Necroptosis	–	–	MLKL	Inhibiting MLKL	NAFLD	Animal experiment	[142]
Necroptosis	–	–	Derlin-1	Via increasing RIPK3-mediated necroptosis	NASH	Animal experiment	[144]
Necroptosis	–	–	ATF3	By inducing expression of RIPK3	NASH	Animal experiment	[145]
Necroptosis	serpina3c	Serine Protease Inhibitors	Foxo1	Down-regulation of the β-catenin/Foxo1/TLR4 signaling pathway	NAFLD	Animal experiment	[146]
Necroptosis	ELA	A dual peroxisome proliferator-activated receptor alpha/delta agonist	PPARα/δ	By activating PPARα and PPAR-δ	NASH	Animal experiment	[147]
Necroptosis	AF6	transmembrane protein	RIPK1	By regulating ubiquitination of RIPK1	NASH	Animal experiment	[148]
Necroptosis		–	UCP1	Inhibition of NK cell necroptosis	NASH	Animal experiment	[149]
Necroptosis	–	–	MLKL	By the activation of TGFβ/Smad 2/3 pathway	NASH	Animal experiment	[150]
Necroptosis	Metformin	A first-line antidiabetic drug	TTP	Via the AMPK-Sirt1 axis	NAFLD	Animal experiment, Vitro experiment	[151]
Pyroptosis	CY-09,MCC950, et al.	NLRP3 inhibitors	NLRP3	By decreasing the accumulation of intracellular lipid droplets	NASH	Clinical practice Phase	[155, 156]
Pyroptosis	Vitamin D	A fat-soluble vitamin	NLRP3	By inhibiting the activation of the NLRP3 inflammasome	NAFLD	Animal experiment	[157]
Pyroptosis	–	–	IRF1	Enhanced PA-Induced AIM2 inflammasome activation and pyroptosis	NAFLD	Animal experiment	[158]
Pyroptosis	MCC950	A specifically inhibitor for NLRP3 inflammasomes	AIM2	By inhibiting the activation of the AIM2 inflammasome	NAFLD	Animal experiment	[158]
Pyroptosis	–	–	GSDMD	By controlling cytokine secretion, NF-ĸB activation, and lipogenesis	NASH	Animal experiment	[153]
Pyroptosis	–	–	caspase-11	Enhanced activation of GSDMD and interleukin-1β	NASH	Animal experiment	[159]
Pyroptosis	VX-765	A inhibitor of Caspase-1	Caspase-1	Via the inhibition of p-STAT3/ANXA2	NASH	Animal experiment, Vitro experiment	[160]
Pyroptosis	BI-1	A inhibitor of Bax	IRE1α	Inhibition of NLRP3 activation	NASH	Animal experiment	[161]
Pyroptosis	–	–	ALDH1B1	By inhibiting the NF-κB pathway activation	NASH	Animal experiment, Vitro experiment	[162]
Pyroptosis	CAV1	An essential regulator of metabolic function	ROS	Via the ROS/TXNIP/NLRP3 pathway	NAFLD	Animal experiment	[163]
Pyroptosis	–	–	p-STAT3	Binding with the promoter of Anxa2 and activation of the NLRP3/caspase-1 inflammasome	NASH	Animal experiment, Vitro experiment	[160]
Pyroptosis	NA	A vitamin used for the treatment of dyslipidemia	TLR4	Inhibiting the formation of NLRP3- apoptosis-associated speck-like protein	NASH	Animal experiment	[164]
Pyroptosis	Ra	An oligosaccharide	Nrf2	Inhibiting the TLR4-MyD88-NF-κB pathway	NAFLD	Animal experiment	[165]
Pyroptosis	–	–	TNF-α	Promoting the activation of the NLRC4 inflammasome	NAFLD	Vitro experiment	[166]
Pyroptosis	–	–	SMS1	Via the activation of DAG-PKCδ-NLRC4 axis	NASH	Animal experiment	[167]
Pyroptosis	PS-ALA	A PS and ALA esterification product	SIRT1	Decreasing the expression of NLRP3 and ASC and reduced the co-localization of NLRP3 and cleave-Caspase-1	NASH	Animal experiment	[168]
Pyroptosis	Taurine	A sulfur-containing β-amino acid	NLRP3	By inhibiting the autophagic-CTSB-NLRP3 inflammasomal pathway	NASH	Animal experiment, Vitro experiment	[169]
Pyroptosis	–	–	circSOD2	By competitively spongeing miR-532-3p, activating the TXNIP/NLRP3 inflammasome signaling pathway	NAFLD	Animal experiment, Vitro experiment	[170]
Pyroptosis	–	–	MIR22HG	Through the miR-9-3p/IGF1 pathway	NASH	Animal experiment, Vitro experiment	[171]
Pyroptosis	GAS5	A sponge of miR-28a-5p	MARCH7	Inhibition of the miR-28a-5p/MARCH7/NLRP3 axis	NAFLD	Animal experiment, Vitro experiment	[172]
Pyroptosis	Antcin A	A small triterpenoid molecule	NLRP3	Inhibiting the assembly and activation of NLRP3 inflammasome	NAFLD	Animal experiment, Vitro experiment	[177]
Pyroptosis	–	–	Caspase-11	By maintaining dual fuel bioenergetics-glycolysis and OXPHOS	NAFLD	Animal experiment	[178]
PANoptosis	Si-Wu-Tang	A traditional Chinese herbal prescription	NOXA	By influencing the intercellular transfer of mtDNA	NAFLD	Animal experiment, Vitro experiment	[181]
PANoptosis	–	–	FNDC4	Activation of AMPKα suppresses PANoptosis	MASLD	Animal experiment, Vitro experiment	[182]
Ferroptosis	–	–	ACSL4	potentiating the ACSL4/p38 MAPK signaling pathway	NAFLD	Animal experiment, Vitro experiment	[194]
Ferroptosis	rosiglitazone and ACSL4 siRNA	Antidiabetic drugs and si RNA	ACSL4	Through diminishing 5-hydroxyeicosatetraenoic acid (5-HETE) content.	NASH	Animal experiment, Vitro experiment	[195]
Ferroptosis	Sodium selenite	A GPX4 activator	GPX4	Elevation of glutathione peroxidase 4 (GPX4) levels	NASH	Animal experiment	[196]
Ferroptosis	–	–	iGPX4	interacts with cGPX4	NAFLD	Vitro experiment, Animal experiment	[197]
Ferroptosis	–	–	TRIM59	Via enhancing GPX4 ubiquitination	NAFLD	Animal experiment, Vitro experiment	[198]
Ferroptosis	–	–	MARCH3	Promoting the ubiquitination and degradation of GPX4	NAFLD	Animal experiment, Vitro experiment	[199]
Ferroptosis	–	–	BMP4	Via upregulation of GPX4	NAFLD/NASH	Animal experiment, Vitro experiment	[200, 201]
Ferroptosis	MUC siRNA	siRNA	MCU	Inhibiting the expression of GPX4 through mtROS-NRF2 pathway, and increased ACSL4 via MCU-ACSL4 interaction	NASH	Animal experiment, Vitro experiment	[202]
Ferroptosis	SPD	A naturally occurring polyamine	ATF4	Upregulation of the ATF4/SLC7A11/GCLM/GPX4 signaling pathway	NAFLD	Vitro experiment	[203]
Ferroptosis		–	ATF4	By inducing SLC7A11 (xCT)	NASH-HCC	Animal experiment	[204]
Ferroptosis	–	–	TFEB	Via nuclear translocation	NASH	Animal experiment	[205]
Ferroptosis	ARB	A glucoside derivative of hydroquinone	FTO	Via inhibiting the FTO/SLC7A11 pathway	NAFLD	Animal experiment, Vitro experiment	[206]
Ferroptosis	–	–	GCN5L1	Acetylating CypD and enhancing its binding with ATP5B	NASH	Animal experiment	[207]
Ferroptosis	PA	A monounsaturated fatty acid	ACSL4	Downregulating ACSL4 and upregulating GPX4 and SLC7A11	NAFLD	Animal experiment	[210]
Ferroptosis	–	–	IGFBP7	Via Ncoa4-mediated ferritinophagy	NAFLD	Animal experiment	[212]
Ferroptosis	–	–	PRDX3	Peroxidized PRDX3 Inhibits cystine uptake	NAFLD	Animal experiment, Vitro experiment	[213]
Ferroptosis	ZEA	A carotenoid found in human serum	p53	By promoting mitochondrial function and inhibiting the p53 pathway	NAFLD	Vitro experiment	[214]
Ferroptosis	–	–	ECH1	Blocking extracellular signal regulated kinases (Erk) pathway	NASH	Animal experiment	[215]
Ferroptosis	rosiglitazone	A medication for the treatment of diabetes mellitus	ACSL4	Through diminishing 5-hydroxyeicosatetraenoic acid	NASH	Animal experiment, Vitro experiment	[195]
Ferroptosis	Liraglutide	A GLP-1 analog	AMPK	Through the activation of AMPK/ACC pathway	NAFLD	Animal experiment, Vitro experiment	[217]
Ferroptosis	Hydrocarbon-stapled peptide	A novel stapled α-helical peptide	EFHD2	Interacting with YWHAZ/14-3-3ζ to control IFNγR2 membrane translocation and IFNγ signaling	NASH	Animal experiment	[221]
Ferroptosis	Cilostazol	An anti-PLT drug	PLT	Prevention of Ectopic Erythrophagocytosis	NAFLD	Animal experiment, Vitro experiment	[222]
Ferroptosis	GCNp-Cur NPs	A Cur loaded nanodelivery system	multiple	Through multiple pathways	NAFLD	Animal experiment, Vitro experiment	[223]
Ferroptosis	DA	A kind of natural tricyclic diterpenoid resin acid	Keap1	Through activating the Keap1/Nrf2-ARE signaling pathway	NAFLD	Animal experiment, Vitro experiment	[255]
Cuproptosis	–	–	FDX1	As a pivotal CRG in both NASH and NASH progression to HCC	NASH	Animal experiment	[229]
Cuproptosis	–	–	CTR1	The Cu/MYC/CTR1 interplay	NAFLD-HCC	Animal experiment	[230]

**Table 3:** Potential therapeutic drugs targeting RCD involved in clinical trials for NAFLD/NASH.

Drug	key target	Putative function chennel	Clinical development	Phase of clinical trial	Disease	Primary endpoint	NCT
Liraglutide	GLP-1R	Pyroptosis and ferroptosis	Recruiting	Phase 4	MAFLD	Liver stiffness and liver steatosis measurement	NCT06501326
Semaglutide	GLP-1R	Pyroptosis and ferroptosis	Not yet recruiting	NA	MAFLD	Liver stiffness and liver steatosis measurement	NCT06764056
Exenatide	GLP-1R	Pyroptosis and ferroptosis	Completed	Phase 2 Phase 3	NASH	Improvement in liver histology	NCT00650546
Survodutide	GLP-1R	Pyroptosis and ferroptosis	Active, not recruiting	Phase 3	NASH	Relative reduction in liver fat content assessed by magnetic resonance imaging-proton density fat fraction (MRI-PDFF)	NCT06309992
Dulaglutide	GLP-1R	Pyroptosis and ferroptosis	Unknown status	Phase 4	NASH	Histological improvement	NCT03648554
IBI362	GLP-1R/GIPR	Pyroptosis and ferroptosis	Not yet recruiting	Phase 3	MAFLD	Percentage change in liver fat content from baseline measured by MRI-PDFF	NCT06884293
Efinopegdutide	GLP-1R/GCGR	Pyroptosis and ferroptosis	Completed	Phase 2	NAFLD	Relative reduction in liver fat content assessed by MRI-PDFF	NCT04944992
Efocipegtrutide	GLP-1R/GIPR/GCGR	Pyroptosis and ferroptosis	Recruiting	Phase 2	NASH	Histopathological improvement	NCT04505436
Licogliflozin	SGLT1/2	Apoptosis and pyroptosis	Terminated	Phase 2	NASH	Improvement in fibrosis without worsening of NASH	NCT04065841
Henagliflozein	SGLT2	Apoptosis and pyroptosis	Recruiting	Phase 4	NAFLD	Liver fat content assessed by MRI-PDFF	NCT06449833
Metformin	AMPK	Pyroptosis and ferroptosis	Completed	Phase 2	NASH	Change in the histological nash activity index	NCT00063232
PXL770	AMPK	Pyroptosis and ferroptosis	Completed	Phase 2	NAFLD	Relative reduction in liver fat content assessed by MRI-PDFF	NCT03763877
Spironolactone	AR	Apoptosis	Completed	Phase 1 Phase 2	NASH in young women	Change in liver stiffness on magnetic resonance elastography	NCT03576755
LPCN 1144	AR	Apoptosis	Completed	Phase 2	NASH in adult men	Absolute change in hepatic fat fraction based on MRI-PDFF measurements	NCT04134091
Testosterone undecanoate	AR	Apoptosis	Completed	Phase 2	NASH in adult men	Severity of steatohepatitis improves	NCT01919294
K-877-ER	PPAR-α	Apoptosis, necroptosis, pyroptosis and ferroptosis	Recruiting	Phase 2	NASH	Improvement in disease activity and no worsening of liver fibrosis	NCT05327127
Pemafibrate	PPAR-α	Apoptosis, necroptosis, pyroptosis and ferroptosis	Active, not recruiting	Phase 2	NAFLD	Change in ALT level	NCT06623539
Pioglitazone	PPAR-γ	Necroptosis	Completed	Phase 4	NASH	Liver histology	NCT00227110
Rosiglitazone	PPAR-γ	Necroptosis	Unknown status	Phase 2	NASH	Liver biopsy histologic improvement	NCT00699036
Aramchol	PPAR-γ	Necroptosis	Completed	Phase 2	HIV-associated NAFLD	Improving hepatic steatosis assessed by MRI	NCT02684591
Elafibranor	PPAR-α/δ	Apoptosis, necroptosis, pyroptosis and ferroptosis	Terminated	Phase 3	NASH	NASH resolution without worsening of fibrosis	NCT02704403
Saroglitazar Magnesium	PPAR-α/γ	Necroptosis, pyroptosis and ferroptosis	Active, not recruiting	Phase 2	NASH	Resolution of steatohepatitis with no worsening of fibrosis	NCT05011305
Lanifibranor	PPARα/δ/γ	Apoptosis, necroptosis, pyroptosis and ferroptosis	Recruiting	Phase 3	NASH	Resolution of NASH and improvement of fibrosis	NCT04849728
Selonsertib	ASK1	Apoptosis	Terminated	Phase 3	NASH	Improvement in fibrosis	NCT03053063
JKB-121	TLR4	Necroptosis and pyroptosis	Completed	Phase 2	NASH	Analysis of MRI-PDFF change	NCT02442687
JKB-122	TLR4	Necroptosis and pyroptosis	Unknown status	Phase 2	NASH	Reduction in NAS and no worsening of fibrosis	NCT04255069
Lovaza	NA	Ferroptosis	Unknown status	Phase 4	NAFLD	Improves fibrosis and the NASH activity index	NCT00941642
MB12066	NQO1	Apoptosis	Terminated	Phase 2	NAFLD	Change in hepatic steatosis	NCT02029586

From the results of both drugs in clinical trials and those approved for market use, it is evident that despite ongoing research and new drug developments, the clinical translation of therapies for NAFLD/NASH faces numerous challenges. In terms of preclinical research, there is currently no NASH preclinical model that can fully replicate the pathological features of human NASH. Humanized liver NASH organoids, liver on a chip models, or the establishment of animal models of NASH based on different etiologies may be potential solutions to address this limitation.

Furthermore, NAFLD/NASH presents significant complexity and progression. It is a multifactorial disease resulting from “multiple hits”, with a complex pathogenesis that is often accompanied by systemic metabolic disorders. Treatments focusing on a single drug or a single mechanism have their drawbacks, which is why multi-targeted therapies using a combination of drugs have emerged as a development direction. Moreover, drawing on comprehensive management approaches from chronic diseases like diabetes (such as personalized exercise and diet plans) may also become a mainstay in the treatment of NAFLD/NASH. Regarding the progression from NAFLD to NASH, liver fibrosis, cirrhosis, and ultimately HCC, different stages of the disease may involve distinct RCD mechanisms in different cell populations, which implies that treatment strategies at each stage may vary. In clinical trials, a critical issue for clinical translation is how to assess the efficacy and timing of drugs targeting RCD across different stages of the disease.

Otherwise, the diversity and complexity of RCD mechanisms must also be considered. As mentioned earlier, RCD involves various forms of cell death, with interactions between different modes of death. These interactions result in suboptimal outcomes when targeting a single pathway. The emergence of PANoptosis demonstrates the potential of targeting multiple RCD pathways, but it is still a relatively recent discovery with mechanisms that are not fully understood and no specific targets identified yet. Similarly, there is limited basic research on cuproptosis in NAFLD/NASH, and the scarcity of preclinical studies hampers subsequent drug development. It is worth noting that RCD pathways are crucial for normal cellular processes and immune regulation. Therefore, selectively targeting PANoptosis or simultaneously targeting multiple RCD pathways without triggering adverse side effects is challenging. Research into liver-specific RCD regulators that can precisely target these pathways may reduce impacts on other tissues, thus minimizing adverse reactions [240].

Finally, patient factors also limit the clinical translation process. NASH exhibits significant heterogeneity among patients, with different individuals potentially showing varying pathological manifestations, complications, and metabolic characteristics. Most NAFLD/NASH clinical trial protocols cannot achieve precision or personalized treatment, which means that single-target therapies may not show consistent effects in clinical trials. The lack of personalized treatment strategies complicates the assessment of drug efficacy and safety, making new drug development more challenging. Therefore, refining patient classification based on individual characteristics during clinical trial recruitment may facilitate subsequent precision and personalized treatment. Additionally, liver biopsy remains the gold standard for NASH clinical diagnosis. The invasiveness of liver biopsy and the randomness of sample collection lead to variability in histopathological assessments. Consequently, ethical concerns and potential treatment risks are unavoidable. It is crucial to develop non-invasive diagnostic technologies to evaluate drug efficacy in the future, thus reducing both risks and ethical issues.

With ongoing research, the potential functions of drugs and their analogs in preclinical studies or clinical trial stages will be explored further and applied in clinical settings. Developing targeted drugs for treating NAFLD/NASH-HCC via RCD modulation and their clinical application holds significant potential.

## Correlation and crosstalk between RCDs

The treatment of NAFLD, NASH, and HCC involves a complex and highly specific process that targets multiple RCD pathways. Numerous RCD pathways can be co-activated by similar signaling events in hepatocytes, and certain molecules may simultaneously influence multiple RCD mechanisms. For example, excessive ROS can induce mitochondrial damage, leading to necrosis, pyroptosis, and ferroptosis. Furthermore, the ferroptosis inhibitor, LPT1, not only inhibits ferroptosis but also disrupts PANoptosis, a pathological process encompassing apoptosis, pyroptosis, and necroptosis. Some shared regulatory nodes among these diverse RCD pathways are delineated in this review (Table 4). In addition to the need for comprehensive investigations into each type of RCD, future research should focus on elucidating the interconnections between these RCD pathways and their potential roles in the progression of liver diseases, thereby improving their application in clinical therapeutic strategies.

**Table 4:** The crosstalk between different RCD pathways in NAFLD/NASH.

Compound /pathway	RCD pathway	Mechanism	Reference
ROS	Apoptosis	Blockade of the oxidative respiratory chain by mitochondrial ROS (mitoROS) kinases can lead to apoptosis.	[259]
	Necroptosis	ROS production is dependent on the key proteins of necroptosis, and mitoROS can regulate necroptosis.	[260]
	Pyroptosis	ROS can activate the NLRP3 inflammasome dependent canonical pyroptosis pathway.	[163]
	Feroptosis	A large amount of ROS depletes the antioxidant GSH, causing GPX4 inactivation and ultimately the occurrence of ferroptosis.	[88]
	Cuproptosis	1. Copper ions generate reactive oxygen species via the Fenton reaction, causing DNA damage, mitochondrial dysfunction, lipid peroxidation, and cell death.	[16, 103]
AMPK	Apoptosis	Phosphorylating the pro-apoptotic caspase-6 protein to inhibit its activation	[178]
	Necroptosis	AMPK-Sirt1 pathway activation in KCs enhances autophagy and reduces hepatocyte necroptosis	[151]
	Feroptosis	Activating AMPK/ACC pathway to reduce ferroptosis	[217]
Nrf-2	Apoptosis	Inhibiting the Nrf-2/HO-1/NQO1 pathway to reduce oxidative stress and apoptosis	[258]
	Pyroptosis	Inhibiting the TLR4-MyD88-NF-κB pathway to reduce Pyroptosis	[165]
	Ferroptosis	activating the Nrf2-ARE signaling pathway to reduce Ferroptosis	[255]
P53	Apoptosis	Inhibiting P53 can suppress apoptosis	[261]
	Feroptosis	Inhibiting the p53/mTOR Pathway to reduce ferroptosis	[262]
Smad2/3	Apoptosis	enhancing the activity of TGF-β1/Smad3 signaling to promote apoptosis	[263]
	Necroptosis	activation of TGFβ/Smad 2/3 pathway promotes Necroptosis	[150]
PRDX3	Apoptosis	maintaining mitochondrial membrane potential, reducing oxidative stress,and reducing apoptosis	[256]
	Feroptosis	Peroxidized PRDX3 inhibits cystine uptake	[213]
	Apoptosis	Removing SUMO from RIPK1 in the TNF-R1 signaling complex (TNF-RSC) can reduce apoptosis	[113]
	Necroptosis	Inhibiting RIPK1 can decrease MLKL activation and Necroptosis	[136]
GSH	Feroptosis	GSH reduces lipid hydroperoxides and thereby protects cells from ferroptosis resulting from the accumulation of lipid peroxides	[90]
	Cuproptosis	Mitochondrial GSH slows down cuproptosis by inhibiting enzyme lipoylation and promoting oligomerization of DLAT	[106]
GPX4	Feroptosis	GPX4 not only regulates ferroptosis but also inhibits pyroptosis by blocking GSDMD cleavage and inhibiting the activity of caspase-11 during inflammasome activation	[264, 265]
	Pyroptosis		
Caspase-8	Apoptosis	1.active caspase-8 can directly activate caspase-3 and caspase-7 to execute exogenous cell apoptosis 2.caspase-8 can inhibit pyroptosis and necroptosis 3.the presence or absence of caspase-8 activity is closely associated with pan-apoptosis	[185]
	Necroptosis		
	Pyroptosis		
	PANoptosis		

## Conclusion and future perspectives

In this review, the significance and feasibility of targeting RCD mechanisms in NAFLD/NASH-HCC therapeutic strategies are evaluated. RCD mechanisms are not only crucial in the pathogenesis and progression of NAFLD/NASH-HCC but also offer innovative therapeutic avenues and strategies, holding considerable clinical potential and promising prospects. Notably, most identified therapeutic targets have only been validated in animal models. Given the complexity of the pathophysiology of human NAFLD/NASH-HCC, which may differ from that of animal models induced by singular dietary factors or isolated stimuli, caution should be exercised when conducting these studies. In addition, current research on targeting RCD through modulation of the gut microbiota and epigenetic modifications to improve NAFLD/NASH remains limited. However, changes in lifestyle and dietary habits can influence both gut microbiota composition and epigenetic status, suggesting that gut microbiota and epigenetic modifications may represent promising directions for future investigation. Furthermore, new RCD mechanisms, including disulfidptosis and alkaliptosis, have yet to be investigated in the context of NAFLD/NASH-HCC [241].

Cell death is the ultimate manifestation and an inevitable phase in the pathological progression of diseases, often serving as an indicator of irreversible tissue damage and marking critical junctures in the transition to advanced disease stages. Manipulating cell death and inflammatory responses for therapeutic intervention is a nuanced process, and research into RCD has significantly improved our understanding of cellular fate regulation. Various RCD pathways, including apoptosis and necroptosis, functionally complement and substitute for each other, implying that targeting a single pathway may prove ineffective. Targeting dual or multiple pathways or intervening with molecules common to different RCD mechanisms (for instance, RIK1) may exhibit significant therapeutic potential in future clinical applications. This approach is consistent with pan-apoptosis and its intervention, further emphasizing the need for research into pan-apoptotic mechanisms to drive breakthroughs in RCD-targeted therapy for NAFLD/NASH-HCC. Moreover, the bidirectional role of RCD at various stages of NAFLD/NASH-HCC must be carefully considered. In NASH progression, RCD inhibition could slow disease progression, whereas promoting RCD may enhance the elimination of malignant cells in HCC. In the advanced stages of NAFLD/NASH, senescent hepatocytes may overexpress GPX4, resisting ferroptosis and persisting within the liver, contributing to the deterioration of hepatic health [242]. Consequently, the implications of inhibiting RCD during the later stages of NASH, such as cirrhosis, remain unclear and may inadvertently exacerbate precancerous changes and promote cancer onset. This highlights the critical need to determine the optimal time window for modulating RCD, which is essential for maximizing the clinical benefits of NAFLD/NASH-HCC therapies. Furthermore, selecting appropriate molecular targets tailored to a specific disease is critical for achieving effective therapeutic outcomes.

The development of new technologies such as CRISPR/Cas9 and single-cell sequencing has opened up more possibilities for understanding the mechanisms of RCD and identifying new therapeutic targets. CRISPR/Cas9, through the knockout of specific RCD-related genes, helps establish specific animal or cell models that simulate the cell death processes in NAFLD/NASH, thereby allowing for the study of hepatocyte injury mechanisms and the effects of drug interventions. Additionally, using CRISPR/Cas9 to perform synthetic lethality screening helps identify key regulatory nodes and clarify the most prominent RCD mechanisms involved in NAFLD/NASH, providing a pathway for discovering new targets. Furthermore, research has already combined CRISPR/Cas9 with lipid nanoparticles to enable the targeted delivery of CRISPR/Cas9 components to specific genes, reducing lipid deposition and hepatic steatosis in NAFLD mouse models [243]. Thus, CRISPR/Cas9 can also assess the feasibility of specific genes as drug targets and develop new targeted strategies. Single-cell sequencing allows us to understand the comprehensive landscape of RCD across different cell subtypes in the liver under the context of NAFLD/NASH, revealing the RCD characteristics of different cell types. Based on this, we can further identify new targets and signaling pathways in specific cell populations. Additionally, combining spatial transcriptomics to map cellular landscapes across different stages of NAFLD/NASH-HCC can elucidate the temporal and spatial activation patterns of RCD pathways at various disease stages. This not only clarifies whether RCD is an initiating factor in disease progression or secondary damage, but also helps define the time window for intervening with RCD in NAFLD/NASH-HCC. Performing single-cell sequencing analysis both before and after drug intervention can determine whether a drug selectively targets specific cell subgroups. Therefore, single-cell sequencing offers unprecedented cellular resolution for targeting RCD in NAFLD/NASH treatment, providing an effective pathway for the realization of precision medicine.

The development of new technologies such as drug design and drug delivery systems also significantly enhances the precision of drug targeting, reduces side effects, and has already demonstrated good outcomes in the treatment of NAFLD/NASH. These advancements have demonstrated promising effects in treating NAFLD/NASH. In the future, drug delivery systems can encapsulate multiple drug components, combining them with specific liver molecules to create synergistic effects that target multiple RCD pathways. Moreover, molecular docking can be used for drug screening, providing a wider range of potential natural compounds for targeted therapies. We hope to develop more personalized and effective treatment options for patients with NAFLD/NASH-HCC, thereby improving their prognosis and quality of life.
